# Gene regulatory network analysis with drug sensitivity reveals synergistic effects of combinatory chemotherapy in gastric cancer

**DOI:** 10.1038/s41598-020-61016-z

**Published:** 2020-03-03

**Authors:** Jeong Hoon Lee, Yu Rang Park, Minsun Jung, Sun Gyo Lim

**Affiliations:** 1 Division of Biomedical Informatics, Seoul National University Biomedical Informatics (SNUBI), Seoul National University College of Medicine, Seoul, 110799 Republic of Korea; 2 Department of Biomedical Systems Informatics, Yonsei University College of Medicine, Seoul, 03722 South Korea; 3 Department of Pathology, Seoul National University College of Medicine, Seoul, 03080 Korea; 4 Department of Gastroenterology, Ajou University School of Medicine, Suwon, 16499 Korea

**Keywords:** Networks and systems biology, Predictive medicine, Predictive markers

## Abstract

The combination of docetaxel, cisplatin, and fluorouracil (DCF) is highly synergistic in advanced gastric cancer. We aimed to explain these synergistic effects at the molecular level. Thus, we constructed a weighted correlation network using the differentially expressed genes between Stage I and IV gastric cancer based on The Cancer Genome Atlas (TCGA), and three modules were derived. Next, we investigated the correlation between the eigengene of the expression of the gene network modules and the chemotherapeutic drug response to DCF from the Genomics of Drug Sensitivity in Cancer (GDSC) database. The three modules were associated with functions related to cell migration, angiogenesis, and the immune response. The eigengenes of the three modules had a high correlation with DCF (−0.41, −0.40, and −0.15). The eigengenes of the three modules tended to increase as the stage increased. Advanced gastric cancer was affected by the interaction the among modules with three functions, namely cell migration, angiogenesis, and the immune response, all of which are related to metastasis. The weighted correlation network analysis model proved the complementary effects of DCF at the molecular level and thus, could be used as a unique methodology to determine the optimal combination of chemotherapy drugs for patients with gastric cancer.

## Introduction

Although its incidence is decreasing in some parts of the world, gastric cancer is still the fourth most common cancer worldwide^1,2^. It poses a critical clinical challenge and is associated with poor prognosis, because a high percentage of patients are diagnosed at an advanced stage in some areas and due to its relatively chemoresistant properties and limited treatment options. Metastatic spread occurs frequently in advanced gastric cancer, and it is one of the major causes of death^3,4^. Systemic chemotherapy, which involves a combination of various cytotoxic agents, constitutes the main type of treatment in the adjuvant or palliative setting for patients with metastatic or recurrent cancer^5–7^. Thus far, the choices regarding chemotherapeutic agents or their combinations have typically been determined according to the results from clinical trials. Therefore, the accurate prediction of the response to combination chemotherapy is labor intensive, time consuming, and expensive because of the explosion in the number of combinations available^8^. Accordingly, a new methodology for conducting a molecular-level analysis in order to understand the association between advanced gastric cancer and chemotherapy sensitivity is required.

Despite the increasing knowledge about tumor biology and pharmacology, our understanding of combination chemotherapy is limited due to the complex factors involved, such as the gene–drug interactions and gene regulatory networks^9^. Nonetheless, various combinations of chemotherapeutic drugs have been extensively tested, because they can increase efficacy, lower dosages, and minimize drug resistance. The combination of docetaxel, cisplatin, and 5-fluorouracil (DCF) is one of the most popular chemotherapy regimens in gastric cancer and is reported to provide a better clinical benefit than each of the agents alone^10–12^. Because of the limited number of studies, it is unclear whether the DCF regimen is the best among the chemotherapeutic agent combinations currently available. We postulated that gene–drug interactions have a critical and significant influence on the efficacy of chemotherapy and that the efficacy of chemotherapy, in various combinations, can be analyzed using a methodology based on these interactions. We believe that, if a methodology is identified to demonstrate the complementary effect of the DCF combination therapy, the candidates for a new drug combination may be indicated by the individual’s genetic profile.

There have been many studies assessing the effect of chemotherapy on cancers using machine learning approach^13–18^. However, investigations into chemotherapeutic drugs, such as 5-fluorouracil and docetaxel, at the molecular level are lacking due to the nature of the cytotoxic drugs^19^. However, with the advent of public data in the form of The Cancer Genome Atlas (TCGA), many molecular-level studies of gastric cancer have been carried out^20^. In addition, the remarkable advances in bioinformatics have facilitated the analysis of these high-throughput data. For example, the differential expression analysis packages DESeq, EdgeR, and limma and the network analysis packages GeneNetWeaver and WGCNA have enabled system-level analyses and the characterization of cancer genomics^21–26^. Drug sensitivity and patient genomic data are linked to inferred synthetic lethal gene pairs^27^. The performance of molecular-based assays is expected to explain the synergistic effects of combination drug therapies. However, no studies have linked TCGA and drug sensitivity databases to infer the synergistic effect of drug combinations, and research into the chemotherapeutic response according to molecular-level analyses is still, to the best of our knowledge, lacking.

In this study, we performed a systematic analysis of the network structure dynamics in conjunction with the chemotherapeutic agents used in early- and advanced-stage gastric cancer patients based on the gastric cancer genome in the TCGA database with large-scale pharmacogenomic profiles of GDSC. We hypothesize that gene regulatory network modules with independent functions in advanced-stage gastric cancer will play an important role in determining not only the prognosis of patients, but also their response to chemotherapeutic agents. Based on the function of each module and the sensitivity of each chemotherapeutic agent, here, our analysis enables us to explain the synergistic effects of DCF at the molecular level.

## Results

### The characteristics of the patients with gastric cancers

The characteristics of 105 gastric cancer patients enrolled from the TCGA are shown in Table 1. The median ages were 71 and 63 year old in stage I and IV, respectively. The most common anatomical site was fundus/body.

**Table 1:** The characteristics of the gastric cancer patients in stage I and IV.

	Stage I	Stage IV	All
**No. of patients**	59	46	105
**Age**	71.00 (62.00, 77.00)	63.00 (54.00, 69.00)	67.00 (58.00, 75.00)
**Status**			
Alive	48	23	71
Dead	11	23	34
**Histological type**			
Stomach adeno. (NOS)	24	8	32
Intestinal adeno. (NOS)	14	13	27
Stomach adeno. diffuse	7	7	14
Stomach intestinal adeno. tubular	9	10	19
Stomach intestinal adeno. mucinous	0	2	2
Stomach intestinal adeno. papillary	3	1	4
Stomach adeno. signet ring	2	4	6
**Anatomic neoplasm subdivision**			
Antrum/distal	14	19	23
Cardia/proximal	10	8	18
Fundus/body	23	12	35
Gastroesophageal junction	8	4	12
Others	3	3	6

### Linkage of differentially expressed genes to the drug sensitivity of cancer cells

**Figure 1:**
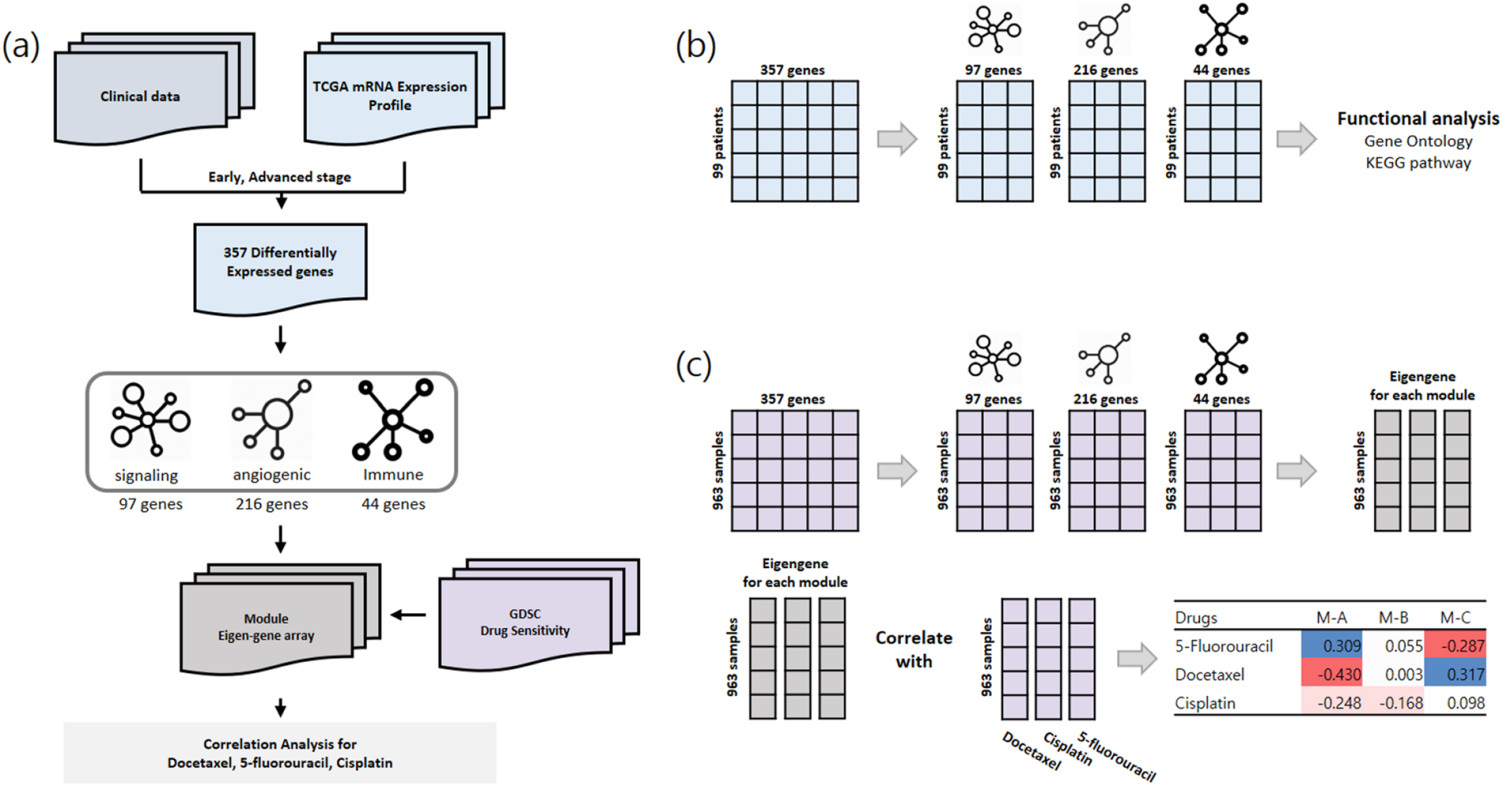
Schematic showing the workflow of the research. (**A**) The entire process used to identify the advanced-stage gastric cancer module and the correlation testing of the module with drug sensitivity. (**B**) Identification of three modules from the differentially expressed genes. Functional enrichment tests were performed for each module. (**C**) The eigengenes for each network were obtained from the COSMIC cell line project expression data. Each module shows a correlation with the sensitivities of 5-fluorouracil, docetaxel, and cisplatin.

We implemented an analysis pipeline to interpret the complementary effects of the drug combinations at the molecular level. The workflow scheme for the entire method is shown in Fig. 1A. Our method is divided into three stages. First, the genes that differentiate between the early- and advanced-stage cancers were identified. Second, the differentially expressed genes were divided into three modules (Fig. 1B). Finally, the eigengenes, which are representative of the expression profile of each module, were calculated and linked to the sensitivities of the drugs (Fig. 1C). We compared the eigengenes of the three modules with the reactivity to the 265 drugs provided in the GDSC database.

### TCGA data processing and differentially expressed genes

TCGA RNA-Seq expression data from 59 early-stage gastric cancer patients and 46 advanced-stage gastric cancer patients were used. From the above threshold of a counts per million (CPM) >1 in more than half of the samples, 6370 genes were excluded from the analysis and 14131 genes remained. We then performed a differential expression analysis on the expression data. Overall, 357 differentially expressed genes were derived (Table S1). Among the differentially expressed genes, 335 were upregulated in advanced-stage gastric cancer patients, and only 22 genes were upregulated in early-stage gastric cancer patients. Subsequently, a functional enrichment analysis was performed on the biological process terms from the Gene Ontology and KEGG databases to determine the types of functions enriched among the differentially expressed genes (Fig. 1B).

### Regulatory network construction and module detection

**Figure 2:**
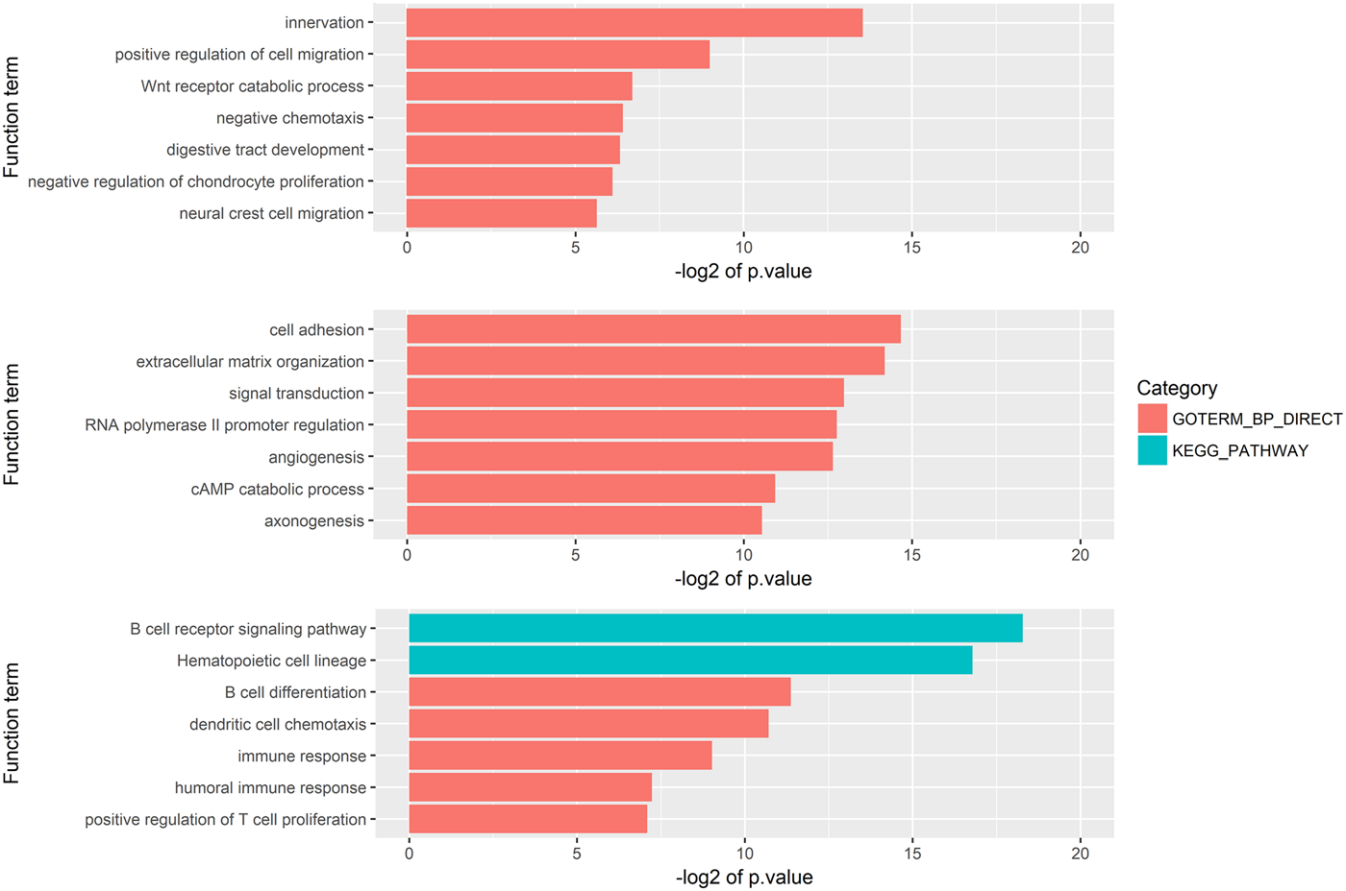
Functional enrichment analysis for each module. A block-wise network construction was performed on the differentially expressed genes and three modules were generated. Each module gene was enriched in biological processes based on the Gene Ontology and KEGG pathway terms. (**A**) First module, related to cell migration and proliferation. (**B**) Second module, related to cell adhesion and angiogenesis. (**C**) Third module, related to the immune response.

For further analysis of the differentially expressed genes, we constructed a block-wise network using the WGCNA package and identified three gene regulatory network modules. The functional enrichment analysis for the biological processes of the Gene Ontology and KEGG pathways was performed for each of the three modules. Module A contained 97 genes that were significantly enriched for functions, such as innervation, cell migration, and catabolic processes (Fig. 2A). Module B contained 216 genes with significant Gene Ontology terms related to cell adhesion, extracellular matrix organization, and angiogenesis (Fig. 2B). Module C contained 44 genes related to Gene Ontology terms that included immune response and B cell differentiation and to the KEGG B cell receptor signaling pathway (Fig. 2C). The three modules clearly had distinct functions.

### Expression patterns of the three modules and responses to anticancer drugs

We hypothesized that the three modules derived from the differentially expressed genes in advanced gastric cancer played a major role not only in determining patient prognosis, but also in the response to chemotherapeutic agents. Therefore, we performed a correlation analysis using the GDSC database drugs and the RNA expression data from the cancer cell lines provided in the GDSC database. The eigengene corresponding to the first principle component of the mRNA expression data in the cell line was determined for the genes belonging to each module (Table S2). Table 2 and Table 3 show the Pearson correlation coefficients between the eigengene values of module A and module C and the IC_50_ values for the chemotherapy drugs, respectively. All of the correlation coefficient results for the 265 drugs and the three modules are available in Supplementary Table 3. The top 50 negatively correlated drugs for each module are shown in Supplementary Table 4.

**Table 2:** Correlation coefficient with the module eigengene for A, B, and C for 12 highly correlated drugs (IC50) for module A.

Drugs	Module A	Module B	Module C
Docetaxel	−0.406	−0.018	0.324
Bleomycin	−0.391	−0.095	0.227
TGX221	−0.385	−0.176	0.131
Tanespimycin	−0.328	0.092	0.355
Dasatinib	−0.299	0.039	0.067
CHIR-99021	−0.276	−0.191	0.064
Piperlongumine	−0.267	−0.145	0.065
RO-3306	−0.265	−0.026	0.189
Elesclomol	−0.262	−0.121	0.156
Trametinib	−0.256	0.096	0.202
XAV939	−0.248	0.078	0.182
Cisplatin	−0.208	−0.151	0.090

**Table 3:** Correlation coefficient with the module eigengene of A, B, and C for 12 highly correlated drugs (IC50) for module C.

Drugs	Module A	Module B	Module C
I-BET-762	0.394	−0.045	−0.560
PIK-93	0.343	−0.036	−0.533
PHA-793887	0.386	0.051	−0.499
AKT inhibitor VIII	0.421	0.069	−0.465
Methotrexate	0.365	0.016	−0.463
NPK76-II-72-1	0.384	−0.030	−0.463
AT-7519	0.347	0.097	−0.449
Vorinostat	0.382	−0.026	−0.428
WZ3105	0.393	0.055	−0.410
5-Fluorouracil	0.342	0.053	−0.404
BMS-345541	0.343	0.046	−0.391
Navitoclax	0.347	−0.065	−0.365

Two patterns in the correlation between the eigengenes of the modules and the drug sensitivity were found. Drugs, such as docetaxel, bleomycin, and cisplatin, had negative correlations with the genes in module A (those related to cell migration and proliferation) (specifically, −0.41, −0.39, and −0.21, respectively), which means that the sensitivity of the chemotherapy increased when the expression of the module A genes increased (Table 2). However, there were positive correlations with the eigengenes of module C (those related to the immune response) (specifically, 0.32, 0.23, and 0.09 for docetaxel, bleomycin, and cisplatin, respectively), which means that the sensitivities of the chemotherapeutic drugs decreased when the expression of the module B genes increased. Conversely, for 5-fluorouracil and methotrexate, module A showed a positive correlation (0.34 and 0.36, respectively), whereas module C showed a negative correlation (−0.40 and −0.46, respectively). However, when the expression level of module C increased, the adjusted AUC value of 5-fluorouracil decreased. Module B (cell adhesion and angiogenesis) was correlated with cisplatin and afatinib, which prevent cancer by blocking new blood vessel formation^28–30^. The range of the correlation coefficients of the drugs in module B was −0.41 to 0.42, which was not as wide as modules A (−0.24, 0.32) and C (−0.56, 0.36).

Drugs with a negative correlation with module A had a positive correlation with module C (Table 2). Likewise, drugs with a negative correlation with module C had a positive correlation with module A. The correlation analyses between module A and the drug sensitivity correlation and module B and the drug sensitivity correlation revealed a correlation coefficient of −0.95, which was a strong negative correlation, indicating that the expression levels of module A and module B were inversely related to the sensitivity of each drug.

### Eigengenes of the three modules according to stage

**Figure 3:**
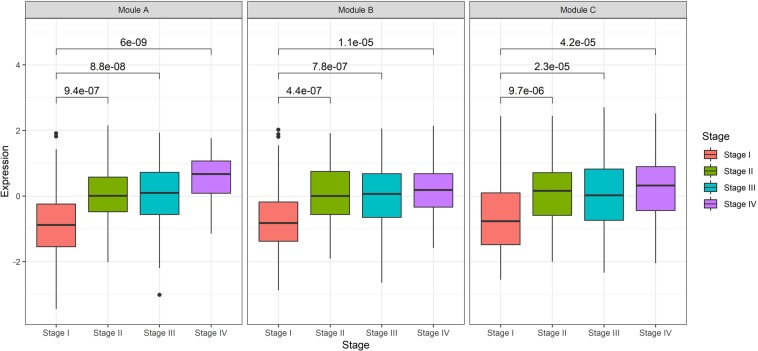
Eigengenes of the three modules composed of the differentially expressed genes between early- and advanced-stage gastric cancer are represented by a boxplot according to stage. The eigengenes of the three modules increased with an increase in the stage, and the expression level of stage I was significantly lower than that of the other stages, based on a Wilcoxon test.

**Figure 4:**
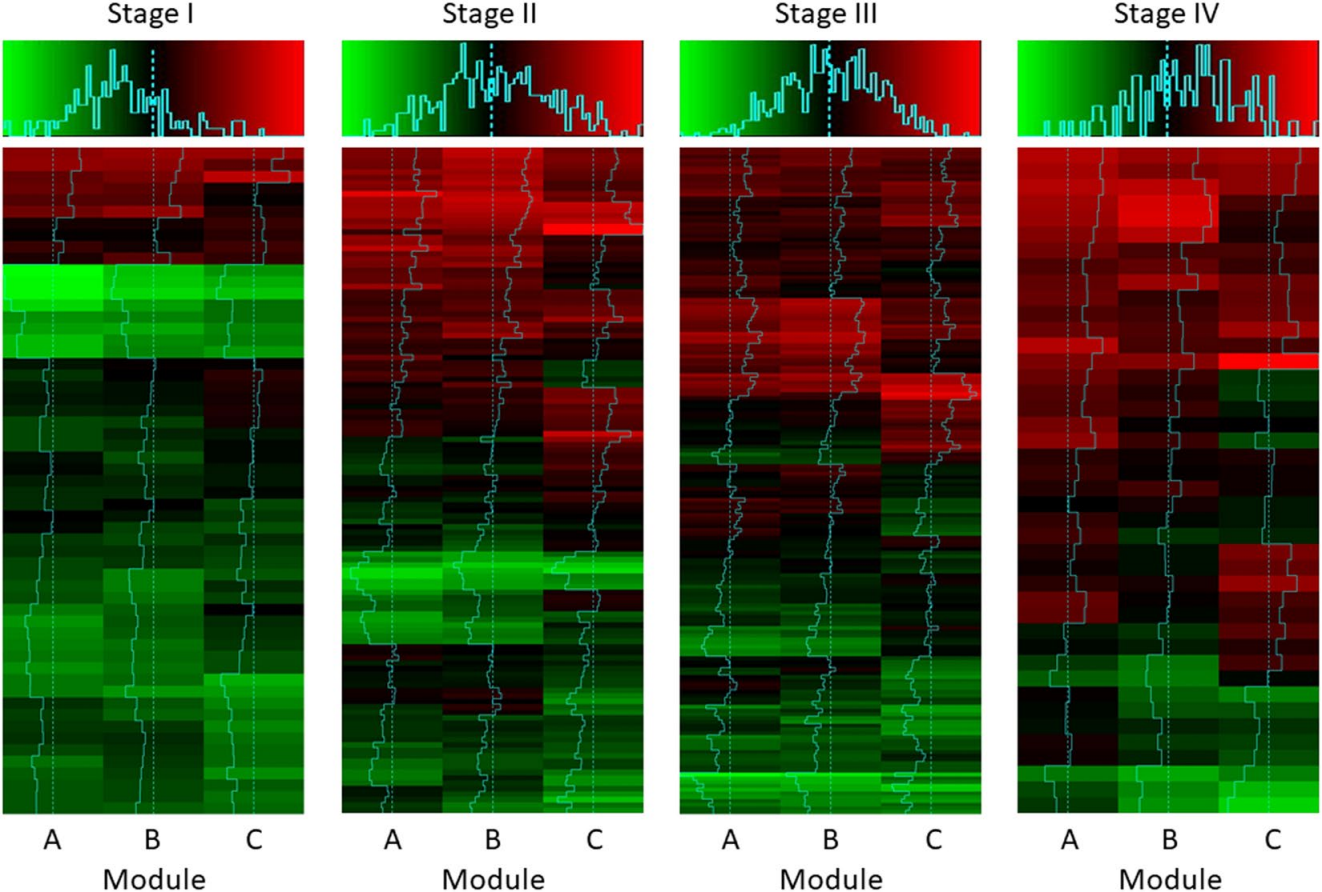
Hierarchical clustering heatmap for the module eigengenes among the patients according to pathologic stage. As the stage increased, the proportion of the patients with a higher value of the module eigengene increased. As the stage increased, the value of the module eigengene became heterogeneous.

We calculated the eigengenes for all of the TCGA patients to determine when the three modules were activated according to stage progression. The stage II patients (122 patients) and stage III patients (177 patients) were added, and the eigengene distribution was plotted as a boxplot according to stage for all 398 patients (Fig. 3). Compared with the stage I patients, the stage II, III, and IV patients had significantly higher eigengene values, indicating that the three modules were activated. The three modules with functions related to cancer metastasis were activated from stage II. Next, hierarchical clustering was performed by dividing the patients according to stage to determine the degree of activation of the three modules for each patient (Fig. 4). Then, a correlation analysis was performed for the eigengenes of each module according to stage. For stage I, the correlation coefficients for the three modules were 0.94, 0.80, and 0.83 in the order of module A and B, module A and C, and module B and C. For stage II, they were 0.92, 0.68, and 0.70, respectively, whereas for stage III, they were 0.92, 0.63, and 0.68, respectively. Finally, for the stage IV patients, the correlation coefficients were 0.88, 0.56, and 0.63, respectively. Thus, as the stage increased, the eigengene values of each module tended to become more heterogeneous.

**Figure 5:**
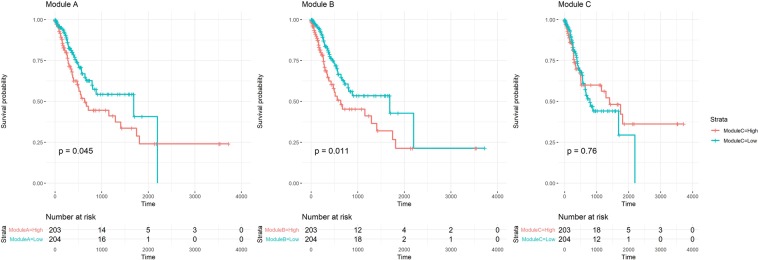
Survival analysis for all 397 patients, including stage II and III patients, was performed to determine whether the eigengenes of the three modules were prognostic factors. According to the median value of the eigengene, the patient group was divided into two groups; the p-values for the log-rank test results are shown. The eigengenes of modules (**A**,**B**) were significant at p < 0.05, whereas that of module (**C**) was not significant.

Stage was strongly associated with survival. For all of the 397 gastric cancer patients from the TCGA, the Cox proportional hazard model p-values for stages II, III, and IV versus stage I were 0.45, 0.21, and <0.01, respectively. Next, a survival analysis was performed to determine whether the eigengene was a prognostic marker for the three modules (Fig. 5). According to the median eigengene value, the patient group was divided into two groups. Log-rank testing determined p-values of 0.05 for module A, 0.01 for module B, and 0.76 for module C. A survival analysis for all 432 gastric cancer patients who were at high risk after curative surgery plus adjuvant chemoradiotherapy was performed to validate whether the eigengenes of modules A and B were prognostic factors in an external dataset (GSE26253) using the expression profile from Illumina HumanRef-8 WG-DASL v3.0. Of the 357 differentially expressed genes, 241 were available. According to the median eigengene value, the entire patient group was divided into two, and the p-values for the log-rank test results were determined. The eigengene of module B was significant at p < 0.05, whereas module C was not significant, which was similar to the TCGA dataset. However, for module A, unlike in the TCGA dataset, survival was not significant (Fig. S1).

## Discussion

In this study, we linked a molecular-level analysis of gastric cancer to chemotherapy sensitivity in order to explain the synergistic effects of DCF in advanced gastric cancer. We constructed a regulatory network based on differentially expressed genes in early- and advanced-stage patients. Using a block-wise network construction in the WCGNA, we derived three modules related to advanced-stage gastric cancer. The three modules were independently associated with cell migration, angiogenesis, and the immune response. Docetaxel had a strong negative correlation with the module related to cell migration and a positive correlation with the module related to the immune response. 5-fluorouracil had a strong correlation with each module but in different directions. Notably, the correlation of 5-fluorouracil with each module was in the opposite direction as those of docetaxel.

Computational models that have been developed to identify candidate antitumoral molecules can be used to predict drug sensitivity and identify synergistic combinations of anti-tumoral chemotherapies^31,32^. Among these, network-based models and machine learning-based models are acknowledged as potent methodologies^33,34^. However, the data volume of the known data is limited and more accurate computational algorithms are needed. To overcome the limitations mentioned above, a combination of using a computational approach, such as network-based and machine learning-based models, and the utilization of heterogenous data sources would provide more excellent outcomes in identifying anti-tumor drugs and their combinations^35,36^.

The three modules identified in this study had significantly independent biological functions (Fig. 2). The module A genes were significantly related to innervation and the positive regulation of cell migration. Module B had genes with significant functions that included cell adhesion, extracellular matrix organization, and angiogenesis. The module C genes were significantly enriched for immune related terms by the Gene Ontology and KEGG pathway analyses. Cell migration, angiogenesis, and the immune response contribute to the process of metastasis. In this study, we also found that the three modules adopted independent characteristics of cancer as the stage progressed. The main function of tumors cells, and thus the hallmarks of cancer, are to sustain proliferative signals, induce angiogenesis, and avoid immune destruction. These three functions could be mapped to the functions of our three modules. In addition, as the stage progressed, the expression levels increased (Fig. 3). The regulation of the expression of the genes belonging to these three modules will be key to preventing the progression to the advanced stage and to stopping metastasis.

Since each module consists of differentially expressed genes in the advanced stage gastric cancer patients, we reviewed the literature to determine which modules were comprised of important cancer genes. Based on this, we created a table that combines the list of Cancer Gene Concensus (CGC) provided by the COSMIC database and the module information of the differentially expressed genes (Supplementary Table 5)^37,38^. Among the CGCs in module A, we identified AKT3, which is an oncogene that is associated with gastric cancer cell proliferation^39^. In addition, we found QKI, which is a tumor suppressor that is associated with cancer prognosis in gastric cancer, and TGFBR2, which is also associated with driver and susceptibility in gastric cancer^40,41^. These genes are the representative oncogenes and tumor suppressor genes from module A that are related to cancer proliferation. Among the CGC genes in module B, we identified PTPRB, which is a tumor suppressor gene that plays an important role in blood vessels and angiogenesis^42^. We also found RNF43, which is also a tumor suppressor gene and signal transducer that inhibits cancer cell proliferation^43^ and was down-regulated in advanced cancer in module B. Among the genes in module C, we found CD28, which is involved in tumor infiltration, size, and lymph node metastasis in gastric cancer^44^. In addition, we identified CXCR4, which plays an important role in the development of peritoneal carcinomatosis from gastric carcinoma^45^. These genes reveal that the function of module C is enriched for immune-related functions.

The eigengenes of the three modules are inter-correlated genes, because they are composed of differentially expressed genes between the early- and advanced-stage patients. However, as the stage progressed, this correlation was lost, and the correlation coefficient tended to decrease. In stage I, the correlation coefficients for modules A and B, A and C, and B and C were 0.94, 0.80, and 0.83, respectively, but they decreased to 0.88, 0.56, and 0.63, respectively, in stage IV. These data indicated that the cancer cells showed a more heterogeneous characteristic as the stage increased (Fig. 4), suggesting that the cancer cell characteristics depended on which of the three modules are activated. Thus, personalized medical treatment can be planned according to the expression pattern of the dominant module of the patient.

Docetaxel and 5-fluorouracil are effective as a combined therapy^5^. Van Cutsem *et al*. compared DCF with cisplatin and fluorouracil (CF), revealing that the time to progression was significantly better for DCF than for CF (log-rank test, p < 0.001), with a 32% risk reduction. In addition, the overall survival was also significantly better with CDF than with CF (p < 0.05). Thus, because of the heterogeneous properties of cancer, combinations of drugs, such as DCF, are more effective than single agents, and various combinatorial therapies have been proposed for advanced gastric cancer.

The genetic characteristics of a cancer appears to underline its susceptibility to chemotherapeutic agents, at least in part. For example, genes regulating transcription or translation are overexpressed in docetaxel-resistance breast cancer, but those related to apoptosis, adhesion, or the cytoskeleton are enriched in docetaxel-sensitive breast cancer^46^. In patients with metastatic gastric carcinoma, genes altered by docetaxel or cisplatin treatment are useful to predict the therapeutic response^47^. Docetaxel, a potent second-generation taxane agent, prevents cell division and promotes apoptosis in gastric cancer, primarily by stabilizing microtubule dynamics^48^. Microtubules are components of the cytoskeleton that are related to cell motility, cell division, development, and signal transition in the neuronal system^49^. Therefore, the sensitive response to docetaxel, as demonstrated in module A, may be associated with microtubule function, as exemplified by some of the GO terms (e.g., innervation, positive regulation of cell migration, and neural crest cell migration). In addition, module B, which was significantly correlated to the responsiveness to cisplatin, was enriched for cell adhesion and extracellular matrix organization. Consistent with this finding, the targeted inhibition of *CDH17*, one of the cadherin molecules, increases apoptosis in gastric cancer in response to cisplatin treatment both *in vivo* and *in vitro*^40^. The enrichment of immune-related gene signatures in module C, which was associated with the 5-fluorouracil response, may indicate that the gastric carcinoma is plagued with numerous tumor-infiltrating lymphocytes, a type of disease that is likely to benefit from adjuvant chemotherapy^50^.

Furthermore, identifying genes related to the chemotherapy response may help to explain the pharmacodynamics underlying the efficacy of the combination treatment. Notably, the sequence of the drugs associated with module A and with module C showed the opposite order (−0.95 correlation). Therefore, the response to docetaxel and 5-fluorouracil may be related to their opposing functions, accounting for the synergistic effect of the docetaxel and 5-fluorouracil combination treatment. Moreover, other chemotherapeutic agents, highly correlated with the same module, can also be investigated through this gene-based approach, particularly other candidate drugs for combinatorial chemotherapy regimens.

In combination chemotherapy, determining the ratio of the drug is also an important issue. As shown in Fig. 4, as the stage progressed, the patient’s transcriptomic profile became more heterogeneous. The eigengenes of each module were also activated differently in the same patient. Because we deduced the effective drugs for each module, we provided a basis for an adjustment to the drug amount depending on the module being activated. This can be regarded as a combination therapy in personalized medicine using genetic profiles and could be used as a backbone model for advanced personalized medicine in the future.

In this study, we linked regulatory network modules to drug response data in order to interpret the synergistic effects of combinatorial chemotherapy at the molecular level. Three differential modules between early and advanced gastric cancer patients were independently associated with functions that play important roles in cancer metastasis. This linkage revealed a relationship between the eigengenes of the three modules and drug sensitivity by linking the TCGA and GDSC database data and explained the synergistic effects of DCF combinatorial chemotherapy. Therefore, this methodology can be used to propose a new candidate combination therapy and as a way to perform precision medicine by controlling the drug dosage according to the patient’s module eigengene.

## Materials and Methods

### Gastric cancer patients and cancer cell line data

We retrieved the clinical information and the RNA expression profiles from the RNA-Seq level 3 dataset from the TCGA (https://cancergenome.nih.gov/)^20^. Based on the pathological stage, 105 patients were included in the analysis. These patients were stage I (n = 59) and stage IV (n = 46), according to the primary solid tumor samples. Accordingly, the RNA-Seq mRNA expression data were classified into two groups, stage I and stage IV.

The Genomics of Drug Sensitivity in Cancer (GDSC) database provides the half maximal inhibitory concentration (IC_50_) values, which are the chemotherapeutic sensitivity values for each cancer cell line^51^. To simplify the preprocessing of the drug response data, we used the pharmacoGx package in R Bioconductor^52,53^. The COSMIC Cancer Cell Line Project provides the mRNA expression profiles of the cancer cell lines, as quantified by the Affymetrix Human Genome U219 mRNA expression array platform, version 83, which are used to measure the response of 265 anticancer drugs in the GDSC^37^. A total of 963 samples were available for both the drug susceptibility data and the gene expression profiles, which were included in the analysis.

### RNA-Seq differential gene expression analysis

To remove the low expression genes, we transformed the scale of the RNA-Seq counts to the CPM on the logarithmic scale using the function ‘cpm’ in the R package edgeR to compare the relative mRNA expressions between the different samples. Only the expression levels of the genes with a CPM >1 in more than half of the samples were used in the subsequent analysis to eliminate the zero count genes. Before performing the differential gene expression analysis, we performed a trimmed mean of M-value (TMM) normalization to estimate the appropriate relative normalization factors that were not affected by outliers and to make the empirical distribution of the transformed mRNA expression closer to the normal distribution using the function ‘voom’ in the limma R package so that a moderate t-test (limma) could be used^54,55^. For the normalized expression data, we applied a linear model for each gene using the lmFit to fit the design based on pathologic stage. An empirical Bayes moderation is carried out by the eBayes function to compute moderated t-statistics and moderated F-statistics and to estimate the log fold changes and standard errors of the differential expression values^23^. The multiple testing FDR method was used for the obtained genes. Finally, the differentially expressed genes were defined using an adjusted p-value of 0.1 and a log-fold-change of 0.5.

### Gene regulatory network construction and module eigengenes

We constructed a regulatory network using the weighted correlation network analysis (WGCNA) for the differentially expressed genes derived from the TCGA data^25^. Firstly, the absolute value of the Pearson correlation was utilized to estimate the distances for all the pairwise gene-gene relationships. Next, a weighted adjacency matrix was constructed using a soft thresholding power adjacency function *a*_*ij*_ = |*cor (x*_*i*_*, x*_*j*_*)|*^β^, where *a*_*ij*_ indicates the weighted Pearson’s correlation coefficient that measures the coexpression distance between gene *i* and gene *j*. We picked an appropriate soft-thresholding power β, which is the lowest integer where the constructed regulatory networks satisfy the approximate scale-free topology, for scale-free topology^56^. The adjacency matrix was used to calculate the Topological overlap matrix (TOM) and the corresponding dissimilarity, which were used to evaluate the direct correlation between the genes and the degree of agreement in association with other genes in the data set^57^. Then, an average linkage hierarchical clustering was performed for the TOM‐based dissimilarity measure. An appropriate minimum gene module size for the dendogram, as derived by the hierarchical clustering, was set to classify the similar genes into one module^58^.

The dimensionality of the two dimensional of module expression profiles was reduced to a single dimension by projecting each sample onto the first principal component. The projection of the module genes onto a principal component can be viewed as a gene-like pattern of expression across samples, called an eigengene. The eigengene patterns of the modules were uncovered by a singular value decomposition (SVD), which was used to perform the principle component analysis (PCA)^59^. The eigengenes, the representative value of the gene expression profiles for the module, were used to represent the cancer expression profiles of each patient.

### Correlation analysis between drug sensitivity and module eigengenes

The GDSC database contains the drug sensitivity between the COSMIC Cell Line Project (CCLP) and anticancer drugs, where the IC_50_ and adjusted AUC values are provided^14,60^. To understand the role of the expression of the gene module for the chemotherapeutic agents, we extracted the gene expression values corresponding to the modules from the COSMIC cell line project data. The expression levels of the gene modules extracted from the 963 cell lines were summarized into the eigengene vector. We performed a Pearson correlation analysis between the IC_50_ values, which were the sensitivity values of the 265 drugs provided by the GDSC and the eigengene of each module, to identify the effective drugs for each module. A low IC_50_ indicated that a small amount of drug killed large number of cancer cells. In other words, a negative correlation between the IC_50_ and the gene expression indicated a sensitive response of the drug.

### Functional enrichment analysis

The functional enrichment analysis was performed on the various gene sets using the DAVID and RDAVIDWebService tool from the Bioconductor repository (https://www.bioconductor) in the R package^61,62^. The Gene Ontology analysis was used to identify the significantly enriched biological process terms, and the KEGG pathway enrichment analysis was also performed^63,64^.

## Supplementary information

Supplementary information
