# Supplementary material for: Gene regulatory network analysis with drug sensitivity reveals synergistic effects of combinatory chemotherapy in gastric cancer
Source: Sci Rep. 2020 Mar 3;10:3932. doi: 10.1038/s41598-020-61016-z (PMC7054272; doi:10.1038/s41598-020-61016-z)

**Gene regulatory network analysis with drug sensitivity reveals synergistic effects of combinatory chemotherapy in gastric cancer**

**(Running Title**: Gene regulatory network for synergistic effect of combinatory chemotherapy)

Jeong Hoon Lee^1^, Yu Rang Park^2^, Minsun Jung^3^, Sun Gyo Lim^4^

^1^Division of Biomedical Informatics, Seoul National University Biomedical Informatics (SNUBI), Seoul National University College of Medicine, Seoul, 110799, Republic of Korea

^2^Department of Biomedical Systems Informatics, Yonsei University College of Medicine, Seoul, 03722, South Korea

^3^Department of Pathology, Seoul National University College of Medicine, Seoul 03080, Korea

^4^Department of Gastroenterology, Ajou University School of Medicine, Suwon 16499, Korea

**Address correspondence to:**

**Sun Gyo Lim, MD**

Department of Gastroenterology, Ajou University School of Medicine,

164, Worldcup-ro, Yeongtong-gu, Suwon, 16499, South Korea

Tel: +82-31-219-6939

Fax: +82-31-219-5999

E-mail: mdlsk75@ajou.ac.kr

**Supplementary table 1**. Differential expression analysis between early-stage gastric cancer patients and 46 advanced-stage gastric cancer patients

| Differentially expressed genes in Module A | | | | | | | | | | | | | | | | | | | | | | | | | |
| --- | --- | --- | --- | --- | --- | --- | --- | --- | --- | --- | --- | --- | --- | --- | --- | --- | --- | --- | --- | --- | --- | --- | --- | --- | --- |
| Gene | | **logFC** | | **AveExpr** | | **t** | | | **P.Value** |  | **Gene** | | | | **logFC** | | | **AveExpr** | | | **t** | | | **P.Value** |  |
| THBS4 | | 2.9923 | | 1.8026 | | 3.8934 | | | 0.0002 |  | **SORCS2** | | | | 1.1266 | | | 1.2728 | | | 3.0686 | | | 0.0028 |  |
| ADH1B | | 2.6706 | | 1.1646 | | 3.9413 | | | 0.0002 |  | **CPE** | | | | 1.1155 | | | 3.8160 | | | 3.6047 | | | 0.0005 |  |
| CHRDL1 | | 2.6651 | | 0.4003 | | 3.7883 | | | 0.0003 |  | **FIBIN** | | | | 1.0858 | | | 2.4766 | | | 3.6116 | | | 0.0005 |  |
| CILP | | 2.3949 | | 1.2962 | | 4.0726 | | | 0.0001 |  | **SETBP1** | | | | 1.0782 | | | 3.2424 | | | 3.1950 | | | 0.0019 |  |
| C7 | | 2.3873 | | 2.5331 | | 3.6029 | | | 0.0005 |  | **MAP6** | | | | 1.0704 | | | 1.1066 | | | 3.0722 | | | 0.0027 |  |
| SFRP2 | | 2.3765 | | 4.6809 | | 3.4716 | | | 0.0008 |  | **ENOX1** | | | | 1.0677 | | | 0.3683 | | | 3.0981 | | | 0.0025 |  |
| SFRP4 | | 2.2657 | | 4.2232 | | 3.6853 | | | 0.0004 |  | **C1S** | | | | 1.0658 | | | 7.3504 | | | 3.6779 | | | 0.0004 |  |
| ABCA8 | | 2.1663 | | 0.7702 | | 3.6773 | | | 0.0004 |  | **RGAG4** | | | | 1.0649 | | | 1.6834 | | | 3.7394 | | | 0.0003 |  |
| PCDHAC2 | | 2.0473 | | 0.1438 | | 4.0007 | | | 0.0001 |  | **GPRASP1** | | | | 1.0574 | | | 1.8376 | | | 3.2559 | | | 0.0015 |  |
| CHRDL2 | | 1.9999 | | 2.3793 | | 3.1950 | | | 0.0019 |  | **IGFBP5** | | | | 1.0486 | | | 8.3409 | | | 3.4160 | | | 0.0009 |  |
| OGN | | 1.9857 | | 2.3274 | | 3.3625 | | | 0.0011 |  | **LUM** | | | | 1.0482 | | | 8.2649 | | | 3.5206 | | | 0.0007 |  |
| SLIT2 | | 1.9164 | | 1.1654 | | 3.5256 | | | 0.0006 |  | **S100B** | | | | 1.0480 | | | 1.0475 | | | 3.1584 | | | 0.0021 |  |
| CPA3 | | 1.8640 | | 2.8802 | | 4.4433 | | | 0.0000 |  | **GUCY1A3** | | | | 1.0477 | | | 5.1316 | | | 3.2459 | | | 0.0016 |  |
| ABCA6 | | 1.8608 | | 0.4205 | | 4.2750 | | | 0.0000 |  | **MAP7D3** | | | | 1.0439 | | | 2.9494 | | | 4.1428 | | | 0.0001 |  |
| SELP | | 1.7967 | | 1.9046 | | 3.8612 | | | 0.0002 |  | **LOC644538** | | | | 1.0412 | | | 1.2566 | | | 3.8601 | | | 0.0002 |  |
| CNR1 | | 1.7763 | | 0.1230 | | 3.1969 | | | 0.0019 |  | **PIK3CG** | | | | 1.0370 | | | 2.5278 | | | 3.2229 | | | 0.0017 |  |
| TPSB2 | | 1.7749 | | 3.5590 | | 4.5132 | | | 0.0000 |  | **ZCCHC24** | | | | 1.0255 | | | 4.6311 | | | 3.5703 | | | 0.0006 |  |
| PRELP | | 1.7711 | | 4.1689 | | 3.5495 | | | 0.0006 |  | **DCN** | | | | 1.0177 | | | 7.7398 | | | 3.5548 | | | 0.0006 |  |
| MFAP5 | | 1.7599 | | 2.6766 | | 3.5595 | | | 0.0006 |  | **P2RY14** | | | | 1.0177 | | | 1.3906 | | | 3.0616 | | | 0.0028 |  |
| GREM1 | | 1.7360 | | 6.8000 | | 3.5950 | | | 0.0005 |  | **ZNF423** | | | | 1.0162 | | | 2.7328 | | | 3.4946 | | | 0.0007 |  |
| LAMA2 | | 1.7006 | | 3.5198 | | 4.4115 | | | 0.0000 |  | **GLI2** | | | | 1.0110 | | | 3.1454 | | | 3.1697 | | | 0.0020 |  |
| RSPO3 | | 1.6749 | | 1.7981 | | 3.4836 | | | 0.0007 |  | **GAS7** | | | | 1.0057 | | | 4.6527 | | | 3.3850 | | | 0.0010 |  |
| PTGER3 | | 1.6675 | | 1.4785 | | 3.6815 | | | 0.0004 |  | **NR2F1** | | | | 1.0042 | | | 3.5946 | | | 3.2131 | | | 0.0018 |  |
| DARC | | 1.6497 | | 2.5440 | | 3.1961 | | | 0.0019 |  | **FLJ42709** | | | | 1.0018 | | | 0.9294 | | | 3.3522 | | | 0.0011 |  |
| AOX1 | | 1.6486 | | 0.5304 | | 3.3291 | | | 0.0012 |  | **SCN4B** | | | | 0.9959 | | | 1.0078 | | | 3.1067 | | | 0.0025 |  |
| DTNA | | 1.6442 | | 1.3240 | | 3.6056 | | | 0.0005 |  | **JAM2** | | | | 0.9959 | | | 2.0053 | | | 3.1438 | | | 0.0022 |  |
| MEOX2 | | 1.6434 | | -0.2619 | | 3.5084 | | | 0.0007 |  | **CDH11** | | | | 0.9944 | | | 5.7240 | | | 3.5114 | | | 0.0007 |  |
| NRXN2 | | 1.6418 | | 0.9383 | | 4.1335 | | | 0.0001 |  | **CMAH** | | | | 0.9930 | | | 2.0018 | | | 3.7644 | | | 0.0003 |  |
| CYP1B1 | | 1.6387 | | 3.2845 | | 3.4972 | | | 0.0007 |  | **CCL2** | | | | 0.9904 | | | 4.5355 | | | 3.2623 | | | 0.0015 |  |
| TPSAB1 | | 1.6294 | | 4.1982 | | 4.0352 | | | 0.0001 |  | **SLC9A9** | | | | 0.9830 | | | 1.8848 | | | 3.0868 | | | 0.0026 |  |
| FMO1 | | 1.6281 | | -0.1442 | | 3.3306 | | | 0.0012 |  | **PDE7B** | | | | 0.9752 | | | 1.6681 | | | 3.1106 | | | 0.0024 |  |
| ABI3BP | | 1.6040 | | 3.2325 | | 3.3537 | | | 0.0011 |  | **TCF21** | | | | 0.9726 | | | 2.8239 | | | 3.4743 | | | 0.0008 |  |
| MS4A2 | | 1.5611 | | 0.4570 | | 4.2324 | | | 0.0001 |  | **LRCH2** | | | | 0.9715 | | | 0.8505 | | | 3.2363 | | | 0.0016 |  |
| GPR133 | | 1.5394 | | 1.4276 | | 3.3403 | | | 0.0012 |  | **SOD3** | | | | 0.9703 | | | 5.6447 | | | 3.3794 | | | 0.0010 |  |
| ADAMTSL3 | | 1.5196 | | 1.4466 | | 3.6493 | | | 0.0004 |  | **CAMK1D** | | | | 0.9647 | | | 1.4445 | | | 3.1348 | | | 0.0023 |  |
| DCLK1 | | 1.5079 | | 1.0578 | | 3.6612 | | | 0.0004 |  | **GPX8** | | | | 0.9603 | | | 4.2183 | | | 4.0610 | | | 0.0001 |  |
| NBEA | | 1.5078 | | 1.5799 | | 3.2539 | | | 0.0016 |  | **TMEM47** | | | | 0.9532 | | | 4.4988 | | | 3.6771 | | | 0.0004 |  |
| FGF7 | | 1.5055 | | 3.2009 | | 3.9572 | | | 0.0001 |  | **FBLN5** | | | | 0.9496 | | | 4.7884 | | | 3.4111 | | | 0.0009 |  |
| GRID1 | | 1.5011 | | 0.2339 | | 4.0969 | | | 0.0001 |  | **PLXDC2** | | | | 0.9489 | | | 3.9726 | | | 3.3049 | | | 0.0013 |  |
| TUB | | 1.4908 | | 0.8578 | | 3.6718 | | | 0.0004 |  | **AKAP2** | | | | 0.9406 | | | 4.8920 | | | 3.5222 | | | 0.0006 |  |
| LRRN2 | | 1.4856 | | 1.0606 | | 3.6495 | | | 0.0004 |  | **ST8SIA1** | | | | 0.9369 | | | 0.8502 | | | 3.3716 | | | 0.0011 |  |
| F13A1 | | 1.4754 | | 3.2833 | | 3.3950 | | | 0.0010 |  | **AEBP1** | | | | 0.9358 | | | 7.6377 | | | 3.0953 | | | 0.0026 |  |
| ANK2 | | 1.4745 | | 2.3622 | | 3.4413 | | | 0.0008 |  | **CFH** | | | | 0.9317 | | | 5.7145 | | | 3.4036 | | | 0.0010 |  |
| RNF150 | | 1.4689 | | 1.0124 | | 3.3112 | | | 0.0013 |  | **SSBP2** | | | | 0.9268 | | | 2.7036 | | | 3.3620 | | | 0.0011 |  |
| LOC399959 | | 1.4573 | | 2.3749 | | 3.5546 | | | 0.0006 |  | **SSPN** | | | | 0.9236 | | | 4.0115 | | | 3.2350 | | | 0.0017 |  |
| MOXD1 | | 1.4489 | | 2.9899 | | 3.6685 | | | 0.0004 |  | **C10orf72** | | | | 0.9150 | | | 3.2086 | | | 3.1138 | | | 0.0024 |  |
| GSTM5 | | 1.4253 | | 0.1571 | | 3.8001 | | | 0.0002 |  | **MCC** | | | | 0.9143 | | | 3.6402 | | | 3.2622 | | | 0.0015 |  |
| PRICKLE1 | | 1.4239 | | 2.3002 | | 4.4645 | | | 0.0000 |  | **SGCE** | | | | 0.9124 | | | 3.4475 | | | 3.1551 | | | 0.0021 |  |
| C1QTNF7 | | 1.4029 | | 0.4195 | | 3.5287 | | | 0.0006 |  | **C1R** | | | | 0.9053 | | | 7.1518 | | | 3.1868 | | | 0.0019 |  |
| ISLR | | 1.3947 | | 5.3293 | | 3.5161 | | | 0.0007 |  | **SRPX2** | | | | 0.8891 | | | 2.9398 | | | 3.2019 | | | 0.0018 |  |
| MAPK10 | | 1.3942 | | 0.7329 | | 3.6792 | | | 0.0004 |  | **LEPR** | | | | 0.8877 | | | 3.3478 | | | 3.4157 | | | 0.0009 |  |
| GLI1 | | 1.3867 | | 1.1808 | | 4.0009 | | | 0.0001 |  | **SNRPN** | | | | 0.8843 | | | 4.0714 | | | 3.1850 | | | 0.0019 |  |
| IL33 | | 1.3697 | | 3.5262 | | 3.4172 | | | 0.0009 |  | **RAB3IL1** | | | | 0.8814 | | | 3.1686 | | | 3.8025 | | | 0.0002 |  |
| RGS4 | | 1.3668 | | 1.9727 | | 3.9579 | | | 0.0001 |  | **PDE4B** | | | | 0.8801 | | | 4.2292 | | | 3.3247 | | | 0.0012 |  |
| RUNX1T1 | | 1.3613 | | 0.9532 | | 3.7496 | | | 0.0003 |  | **LOC283070** | | | | 0.8781 | | | 2.6202 | | | 3.3949 | | | 0.0010 |  |
| SRPX | | 1.3561 | | 2.3051 | | 3.8328 | | | 0.0002 |  | **A2M** | | | | 0.8754 | | | 9.0215 | | | 3.6658 | | | 0.0004 |  |
| PDGFRL | | 1.3530 | | 0.9236 | | 3.2143 | | | 0.0018 |  | **RECK** | | | | 0.8750 | | | 2.7182 | | | 3.2441 | | | 0.0016 |  |
| SPON1 | | 1.3419 | | 4.9683 | | 3.1486 | | | 0.0022 |  | **MID2** | | | | 0.8705 | | | 3.6110 | | | 3.0967 | | | 0.0025 |  |
| ROR2 | | 1.3394 | | 2.8781 | | 3.2044 | | | 0.0018 |  | **ANTXR1** | | | | 0.8676 | | | 6.8743 | | | 3.0659 | | | 0.0028 |  |
| FAM198A | | 1.3383 | | 0.1426 | | 3.0729 | | | 0.0027 |  | **ZNF521** | | | | 0.8599 | | | 2.9426 | | | 3.3984 | | | 0.0010 |  |
| CD163L1 | | 1.3307 | | 1.8212 | | 4.0007 | | | 0.0001 |  | **TM6SF1** | | | | 0.8590 | | | 1.4687 | | | 3.3273 | | | 0.0012 |  |
| KIAA1644 | | 1.3243 | | 1.4415 | | 3.2625 | | | 0.0015 |  | **MEF2C** | | | | 0.8535 | | | 4.4398 | | | 3.8047 | | | 0.0002 |  |
| NPTXR | | 1.3239 | | 2.3365 | | 3.4166 | | | 0.0009 |  | **GEM** | | | | 0.8525 | | | 4.8686 | | | 3.1085 | | | 0.0025 |  |
| COLEC12 | | 1.3194 | | 2.2976 | | 3.3396 | | | 0.0012 |  | **ARMCX1** | | | | 0.8511 | | | 2.2025 | | | 3.2822 | | | 0.0014 |  |
| HMCN1 | | 1.3153 | | 3.2049 | | 3.3379 | | | 0.0012 |  | **KLF12** | | | | 0.8473 | | | 3.8056 | | | 3.2384 | | | 0.0016 |  |
| MGP | | 1.3020 | | 6.2754 | | 3.5967 | | | 0.0005 |  | **GLT8D2** | | | | 0.8461 | | | 2.8054 | | | 3.1898 | | | 0.0019 |  |
| SSC5D | | 1.2985 | | 3.5625 | | 3.3300 | | | 0.0012 |  | **ZEB1** | | | | 0.8449 | | | 5.1807 | | | 3.1961 | | | 0.0019 |  |
| CCDC80 | | 1.2971 | | 5.4868 | | 3.1083 | | | 0.0025 |  | **AKT3** | | | | 0.8389 | | | 4.2210 | | | 3.1813 | | | 0.0020 |  |
| PDE1A | | 1.2918 | | 1.6605 | | 4.0950 | | | 0.0001 |  | **CTSK** | | | | 0.8384 | | | 5.7043 | | | 3.4495 | | | 0.0008 |  |
| TMEM90B | | 1.2907 | | 1.0231 | | 3.0925 | | | 0.0026 |  | **C14orf37** | | | | 0.8321 | | | 1.2255 | | | 3.4219 | | | 0.0009 |  |
| MFAP4 | | 1.2806 | | 5.7748 | | 3.0897 | | | 0.0026 |  | **TIMP2** | | | | 0.8287 | | | 8.1366 | | | 3.4880 | | | 0.0007 |  |
| FOLR2 | | 1.2695 | | 2.4762 | | 3.3287 | | | 0.0012 |  | **ZEB2** | | | | 0.8264 | | | 4.9309 | | | 3.5136 | | | 0.0007 |  |
| SVEP1 | | 1.2689 | | 3.5223 | | 3.5270 | | | 0.0006 |  | **KCNJ8** | | | | 0.8255 | | | 2.8821 | | | 3.2594 | | | 0.0015 |  |
| NTM | | 1.2683 | | 1.8566 | | 3.2206 | | | 0.0017 |  | **OLFML1** | | | | 0.8109 | | | 3.0256 | | | 3.4110 | | | 0.0009 |  |
| BICC1 | | 1.2636 | | 2.1807 | | 3.2743 | | | 0.0015 |  | **C16orf45** | | | | 0.8088 | | | 3.2629 | | | 3.0581 | | | 0.0029 |  |
| INMT | | 1.2618 | | 1.9080 | | 3.5636 | | | 0.0006 |  | **FAM26E** | | | | 0.8077 | | | 2.1166 | | | 3.1566 | | | 0.0021 |  |
| PRKAR2B | | 1.2487 | | 3.0023 | | 3.7114 | | | 0.0003 |  | **GGT5** | | | | 0.8066 | | | 4.7184 | | | 3.1219 | | | 0.0024 |  |
| PODN | | 1.2422 | | 4.7235 | | 3.2812 | | | 0.0014 |  | **NR3C1** | | | | 0.7833 | | | 5.2228 | | | 3.5760 | | | 0.0005 |  |
| P2RX1 | | 1.2379 | | 0.8072 | | 3.9431 | | | 0.0001 |  | **RCAN2** | | | | 0.7768 | | | 3.6380 | | | 3.2350 | | | 0.0017 |  |
| CCDC136 | | 1.2377 | | 0.7676 | | 3.0792 | | | 0.0027 |  | **GLIS2** | | | | 0.7715 | | | 4.8700 | | | 3.2060 | | | 0.0018 |  |
| EFEMP1 | | 1.2342 | | 5.1074 | | 3.5481 | | | 0.0006 |  | **ECM2** | | | | 0.7669 | | | 2.9667 | | | 3.0679 | | | 0.0028 |  |
| CH25H | | 1.2320 | | 1.3632 | | 3.2538 | | | 0.0016 |  | **JAM3** | | | | 0.7612 | | | 4.3532 | | | 3.2382 | | | 0.0016 |  |
| ABCC9 | | 1.2289 | | 3.0596 | | 3.3701 | | | 0.0011 |  | **PALM2-AKAP2** | | | | 0.7600 | | | 5.4430 | | | 3.9131 | | | 0.0002 |  |
| CNTN4 | | 1.2243 | | 1.4664 | | 3.5574 | | | 0.0006 |  | **EBF1** | | | | 0.7592 | | | 2.9774 | | | 3.3341 | | | 0.0012 |  |
| ZFPM2 | | 1.2211 | | 1.4039 | | 3.5479 | | | 0.0006 |  | **BASP1** | | | | 0.7586 | | | 4.0831 | | | 3.3335 | | | 0.0012 |  |
| LOC339524 | | 1.2116 | | 0.3554 | | 3.6872 | | | 0.0004 |  | **MAGEH1** | | | | 0.7527 | | | 2.7756 | | | 3.4937 | | | 0.0007 |  |
| LSAMP | | 1.2070 | | 1.8324 | | 3.2721 | | | 0.0015 |  | **LOC728392** | | | | 0.7467 | | | 1.5922 | | | 3.1296 | | | 0.0023 |  |
| PRKD1 | | 1.2016 | | 1.3230 | | 3.5287 | | | 0.0006 |  | **RBMS1** | | | | 0.7312 | | | 5.0305 | | | 3.3620 | | | 0.0011 |  |
| CDH23 | | 1.1988 | | 0.3319 | | 3.2547 | | | 0.0016 |  | **CNRIP1** | | | | 0.7307 | | | 2.7391 | | | 3.3752 | | | 0.0011 |  |
| NAV3 | | 1.1962 | | 0.3825 | | 3.3263 | | | 0.0012 |  | **CALHM2** | | | | 0.7306 | | | 3.7115 | | | 3.9873 | | | 0.0001 |  |
| PKIA | | 1.1864 | | 1.0956 | | 4.0746 | | | 0.0001 |  | **MDFIC** | | | | 0.7203 | | | 4.3572 | | | 3.1111 | | | 0.0024 |  |
| FLRT2 | | 1.1773 | | 2.7810 | | 3.4437 | | | 0.0008 |  | **FAM171B** | | | | 0.7161 | | | 2.6629 | | | 3.2092 | | | 0.0018 |  |
| VGLL3 | | 1.1694 | | 2.9787 | | 3.5305 | | | 0.0006 |  | **CLIC2** | | | | 0.7085 | | | 3.0839 | | | 3.2910 | | | 0.0014 |  |
| LPPR4 | | 1.1636 | | 1.6424 | | 3.6252 | | | 0.0005 |  | **LDB2** | | | | 0.6944 | | | 3.1605 | | | 3.2785 | | | 0.0014 |  |
| RBMS3 | | 1.1631 | | 0.4492 | | 3.4357 | | | 0.0009 |  | **NFATC1** | | | | 0.6935 | | | 4.1238 | | | 3.2709 | | | 0.0015 |  |
| PDLIM3 | | 1.1602 | | 5.1073 | | 3.1867 | | | 0.0019 |  | **VIM** | | | | 0.6844 | | | 9.1642 | | | 3.2770 | | | 0.0014 |  |
| GHR | | 1.1593 | | 1.0358 | | 3.4674 | | | 0.0008 |  | **QKI** | | | | 0.6832 | | | 5.7644 | | | 3.1613 | | | 0.0021 |  |
| ARHGAP20 | | 1.1558 | | 0.6205 | | 3.3028 | | | 0.0013 |  | **GADD45B** | | | | 0.6824 | | | 4.6036 | | | 3.0819 | | | 0.0027 |  |
| C7orf58 | | 1.1552 | | 3.4895 | | 3.1565 | | | 0.0021 |  | **UBE2E2** | | | | 0.6798 | | | 3.2791 | | | 3.0690 | | | 0.0028 |  |
| SYNE1 | | 1.1507 | | 4.8564 | | 3.5902 | | | 0.0005 |  | **CD200** | | | | 0.6726 | | | 3.0443 | | | 3.0886 | | | 0.0026 |  |
| HGF | | 1.1505 | | 1.3921 | | 3.1410 | | | 0.0022 |  | **ERG** | | | | 0.6597 | | | 3.5074 | | | 3.2280 | | | 0.0017 |  |
| THSD7A | | 1.1458 | | 0.7138 | | 3.5615 | | | 0.0006 |  | **NRP1** | | | | 0.6488 | | | 6.3372 | | | 3.3690 | | | 0.0011 |  |
| EVC2 | | 1.1456 | | 0.6027 | | 3.2753 | | | 0.0015 |  | **VEGFC** | | | | 0.6407 | | | 2.5733 | | | 3.1954 | | | 0.0019 |  |
| PDE1B | | 1.1384 | | 1.3544 | | 3.5219 | | | 0.0006 |  | **RFTN1** | | | | 0.6311 | | | 4.6818 | | | 3.0566 | | | 0.0029 |  |
| CORO2B | | 1.1364 | | 0.4768 | | 3.7220 | | | 0.0003 |  | **SEPT4** | | | | 0.6257 | | | 2.3779 | | | 3.1184 | | | 0.0024 |  |
| MEOX1 | | 1.1353 | | 0.9568 | | 3.1381 | | | 0.0022 |  | **PKD2** | | | | 0.6040 | | | 4.7478 | | | 3.1131 | | | 0.0024 |  |
| CLIP4 | | 1.1339 | | 3.0582 | | 3.5004 | | | 0.0007 |  | **TGFBR2** | | | | 0.5852 | | | 7.4008 | | | 3.1956 | | | 0.0019 |  |
| C2orf74 | | 1.1324 | | 1.2383 | | 3.0972 | | | 0.0025 |  | **LRRC8C** | | | | 0.5785 | | | 4.5062 | | | 3.2944 | | | 0.0014 |  |
|  |  | |  | |  | |  |  | | | |  | |  | | |  | | |  | | |  |  |  |
| Differentially expressed genes in Module B | | | | | | | | | | | | | | | | | | | | | | | | |  |
| Gene | | **logFC** | | **AveExpr** | | **t** | | | **P.Value** |  | **Gene** | | | | **logFC** | | | **AveExpr** | | | **t** | | | **P.Value** |  |
| TFF2 | | 2.5401 | | 3.7865 | | 3.1041 | | | 0.0025 |  | **MAMLD1** | | | | 1.0120 | | | 2.5155 | | | 4.1069 | | | 0.0001 |  |
| COMP | | 2.1572 | | 1.3397 | | 3.1647 | | | 0.0021 |  | **AADAT** | | | | 1.0029 | | | 1.9362 | | | 3.2531 | | | 0.0016 |  |
| APOD | | 2.1402 | | 4.2823 | | 3.9949 | | | 0.0001 |  | **GSPT2** | | | | 0.9924 | | | 1.6764 | | | 3.2974 | | | 0.0014 |  |
| SEMA3E | | 2.1237 | | 0.3059 | | 3.3144 | | | 0.0013 |  | **NNMT** | | | | 0.9883 | | | 5.1226 | | | 3.3999 | | | 0.0010 |  |
| KIAA1324L | | 1.9882 | | 1.8432 | | 3.9641 | | | 0.0001 |  | **FAM20A** | | | | 0.9779 | | | 2.2676 | | | 3.1284 | | | 0.0023 |  |
| VSTM2L | | 1.8980 | | 0.4460 | | 4.0833 | | | 0.0001 |  | **ZNF542** | | | | 0.9750 | | | 2.1794 | | | 3.3239 | | | 0.0012 |  |
| L1CAM | | 1.8497 | | 2.0079 | | 3.3924 | | | 0.0010 |  | **EMB** | | | | 0.9584 | | | 5.4504 | | | 3.5615 | | | 0.0006 |  |
| SEMA3D | | 1.8162 | | 0.2018 | | 3.5276 | | | 0.0006 |  | **ADORA1** | | | | 0.9576 | | | 0.4265 | | | 3.1169 | | | 0.0024 |  |
| FMO2 | | 1.7751 | | 1.6932 | | 3.1102 | | | 0.0024 |  | **MRAS** | | | | 0.9357 | | | 3.7942 | | | 3.6556 | | | 0.0004 |  |
| COL4A4 | | 1.7618 | | 1.6784 | | 4.0762 | | | 0.0001 |  | **C6orf204** | | | | 0.8987 | | | 1.0302 | | | 3.3856 | | | 0.0010 |  |
| BMPR1B | | 1.7512 | | 0.0034 | | 3.2944 | | | 0.0014 |  | **LOC647121** | | | | 0.8987 | | | 1.3108 | | | 3.0938 | | | 0.0026 |  |
| C20orf103 | | 1.7443 | | 0.3585 | | 3.9941 | | | 0.0001 |  | **IGFBP3** | | | | 0.8866 | | | 7.4674 | | | 3.5100 | | | 0.0007 |  |
| CARD11 | | 1.6731 | | 3.9184 | | 4.6632 | | | 0.0000 |  | **CPNE8** | | | | 0.8828 | | | 2.5387 | | | 3.2983 | | | 0.0013 |  |
| UST | | 1.6708 | | 2.4407 | | 4.3615 | | | 0.0000 |  | **FHAD1** | | | | 0.8734 | | | 0.6979 | | | 3.1285 | | | 0.0023 |  |
| KCNJ12 | | 1.6003 | | 0.1283 | | 3.7105 | | | 0.0003 |  | **SCG5** | | | | 0.8676 | | | 1.1297 | | | 3.3547 | | | 0.0011 |  |
| NOV | | 1.5857 | | 2.3621 | | 5.0048 | | | 0.0000 |  | **SULF2** | | | | 0.8403 | | | 7.2035 | | | 3.5515 | | | 0.0006 |  |
| ACSS3 | | 1.5729 | | 0.2172 | | 3.9346 | | | 0.0002 |  | **ST3GAL6** | | | | 0.8254 | | | 1.3566 | | | 3.4389 | | | 0.0009 |  |
| RET | | 1.4989 | | 0.5567 | | 3.2319 | | | 0.0017 |  | **GATA2** | | | | 0.7684 | | | 2.5435 | | | 3.0851 | | | 0.0026 |  |
| KIT | | 1.4114 | | 2.9628 | | 4.0049 | | | 0.0001 |  | **GALNTL4** | | | | 0.7566 | | | 3.0100 | | | 3.4580 | | | 0.0008 |  |
| SERPINE2 | | 1.3932 | | 4.5998 | | 4.2437 | | | 0.0000 |  | **DSE** | | | | 0.7560 | | | 4.7085 | | | 4.0571 | | | 0.0001 |  |
| SHISA2 | | 1.3895 | | 1.7126 | | 3.8106 | | | 0.0002 |  | **LBH** | | | | 0.7436 | | | 5.5764 | | | 3.7348 | | | 0.0003 |  |
| SNCAIP | | 1.3595 | | 1.5465 | | 3.6977 | | | 0.0004 |  | **FAM164A** | | | | 0.7169 | | | 3.1639 | | | 3.3201 | | | 0.0013 |  |
| FST | | 1.3547 | | 0.4845 | | 3.2010 | | | 0.0018 |  | **PTPRB** | | | | 0.6452 | | | 5.2211 | | | 3.4511 | | | 0.0008 |  |
| SCG2 | | 1.3484 | | 0.7664 | | 3.6675 | | | 0.0004 |  | **L3MBTL3** | | | | 0.5835 | | | 3.0553 | | | 3.0817 | | | 0.0027 |  |
| CCL11 | | 1.3296 | | 2.5208 | | 3.2334 | | | 0.0017 |  | **TMEM86A** | | | | 0.5198 | | | 2.7430 | | | 3.2072 | | | 0.0018 |  |
| EFNA5 | | 1.2868 | | 2.4357 | | 3.3506 | | | 0.0011 |  | **TMEM133** | | | | 0.5188 | | | 2.9953 | | | 3.0567 | | | 0.0029 |  |
| NELL2 | | 1.2631 | | 0.4120 | | 3.1639 | | | 0.0021 |  | **PARP10** | | | | -0.5054 | | | 5.8558 | | | -3.2539 | | | 0.0016 |  |
| TDO2 | | 1.2489 | | 0.9259 | | 3.5109 | | | 0.0007 |  | **NECAB3** | | | | -0.5161 | | | 4.5041 | | | -3.7041 | | | 0.0003 |  |
| GFPT2 | | 1.2321 | | 2.3956 | | 3.2744 | | | 0.0015 |  | **PPP1R16A** | | | | -0.5230 | | | 5.7045 | | | -3.2193 | | | 0.0017 |  |
| HSD11B1 | | 1.2310 | | 0.8751 | | 3.1478 | | | 0.0022 |  | **FAM115A** | | | | -0.5393 | | | 3.7524 | | | -3.3590 | | | 0.0011 |  |
| RIMKLB | | 1.2164 | | 2.6994 | | 3.6808 | | | 0.0004 |  | **GSR** | | | | -0.5425 | | | 6.0304 | | | -3.5928 | | | 0.0005 |  |
| C11orf63 | | 1.2164 | | 0.7133 | | 3.9336 | | | 0.0002 |  | **SCRIB** | | | | -0.5474 | | | 7.0495 | | | -3.1216 | | | 0.0024 |  |
| LPL | | 1.1938 | | 2.7221 | | 3.7940 | | | 0.0003 |  | **REPIN1** | | | | -0.5694 | | | 7.7259 | | | -3.8668 | | | 0.0002 |  |
| PTPN13 | | 1.1894 | | 2.7229 | | 3.2329 | | | 0.0017 |  | **ACTR3C** | | | | -0.5805 | | | 1.5163 | | | -3.0943 | | | 0.0026 |  |
| CD180 | | 1.1893 | | 2.2370 | | 3.6329 | | | 0.0004 |  | **DUSP16** | | | | -0.5815 | | | 5.8851 | | | -3.6644 | | | 0.0004 |  |
| RNASE1 | | 1.1798 | | 7.2148 | | 4.7004 | | | 0.0000 |  | **SLC25A37** | | | | -0.5841 | | | 5.0666 | | | -4.0284 | | | 0.0001 |  |
| PTHLH | | 1.1771 | | 0.5092 | | 3.3568 | | | 0.0011 |  | **FAM83G** | | | | -0.6145 | | | 4.7266 | | | -3.4299 | | | 0.0009 |  |
| EOMES | | 1.1740 | | 0.6529 | | 3.1554 | | | 0.0021 |  | **TIGD5** | | | | -0.6265 | | | 3.8007 | | | -3.6586 | | | 0.0004 |  |
| PELI2 | | 1.0936 | | 4.0598 | | 4.2734 | | | 0.0000 |  | **ADCK5** | | | | -0.6335 | | | 3.6876 | | | -4.0466 | | | 0.0001 |  |
| EDA | | 1.0881 | | 1.6686 | | 3.4802 | | | 0.0007 |  | **AMACR** | | | | -0.6769 | | | 5.0388 | | | -3.1139 | | | 0.0024 |  |
| KCNS3 | | 1.0760 | | 2.9186 | | 3.8936 | | | 0.0002 |  | **PPFIA3** | | | | -0.7194 | | | 3.7097 | | | -3.4648 | | | 0.0008 |  |
| ENPP2 | | 1.0722 | | 4.3901 | | 3.5970 | | | 0.0005 |  | **ZNRF3** | | | | -0.7280 | | | 4.8220 | | | -3.0692 | | | 0.0028 |  |
| CDK14 | | 1.0672 | | 3.4536 | | 3.9052 | | | 0.0002 |  | **LOC113230** | | | | -0.8495 | | | 4.2602 | | | -3.2802 | | | 0.0014 |  |
| PAQR5 | | 1.0519 | | 2.8303 | | 3.1446 | | | 0.0022 |  | **ZNF703** | | | | -0.9185 | | | 6.5632 | | | -3.1741 | | | 0.0020 |  |
| RAB37 | | 1.0487 | | 1.4109 | | 3.7848 | | | 0.0003 |  | **SLC5A1** | | | | -1.3127 | | | 4.9182 | | | -3.1290 | | | 0.0023 |  |
| UNC13A | | 1.0436 | | 0.5809 | | 3.3036 | | | 0.0013 |  | **RNF43** | | | | -1.3279 | | | 5.7017 | | | -4.1565 | | | 0.0001 |  |
| HRH2 | | 1.0429 | | 0.3210 | | 3.5970 | | | 0.0005 |  | **ASCL2** | | | | -1.4842 | | | 3.7257 | | | -3.0713 | | | 0.0027 |  |
| NMNAT2 | | 1.0405 | | 1.4070 | | 3.0915 | | | 0.0026 |  | **SP5** | | | | -1.5811 | | | 2.0571 | | | -3.5541 | | | 0.0006 |  |
| ALDH1A3 | | 1.0267 | | 4.3354 | | 3.2141 | | | 0.0018 |  |  | | | |  | | |  | | |  | | |  |  |
|  | |  | |  | |  | | |  |  |  | |  | | |  | | |  | | |  |  |  |  |
| Differentially expressed genes in Module C | | | | | | | | | | | | | | | | | | | | | | | | |  |
| Gene | | **logFC** | | **AveExpr** | | **t** | | | **P.Value** |  | **Gene** | | | | **logFC** | | | **AveExpr** | | | **t** | | | **P.Value** |  |
| CR2 | | 2.2479 | | 0.8174 | | 3.4635 | | | 0.0008 |  | **CD37** | | | | 0.9604 | | | 4.2828 | | | 3.2516 | | | 0.0016 |  |
| CCL19 | | 2.1881 | | 0.5885 | | 3.5980 | | | 0.0005 |  | **CXCR4** | | | | 0.9594 | | | 5.8939 | | | 3.8410 | | | 0.0002 |  |
| MS4A1 | | 1.8408 | | 0.9576 | | 3.2490 | | | 0.0016 |  | **SFMBT2** | | | | 0.9501 | | | 1.2241 | | | 3.1963 | | | 0.0019 |  |
| CD22 | | 1.7499 | | 1.0880 | | 4.3433 | | | 0.0000 |  | **FAIM3** | | | | 0.9303 | | | 3.0603 | | | 3.1238 | | | 0.0023 |  |
| CR1 | | 1.5579 | | 1.2358 | | 3.4953 | | | 0.0007 |  | **RNASE6** | | | | 0.8887 | | | 3.5462 | | | 3.2543 | | | 0.0016 |  |
| CD1C | | 1.3892 | | -0.2352 | | 3.2476 | | | 0.0016 |  | **CXorf21** | | | | 0.8829 | | | 0.5415 | | | 3.4565 | | | 0.0008 |  |
| CCR2 | | 1.3707 | | 0.4248 | | 3.7072 | | | 0.0003 |  | **PIK3R6** | | | | 0.8577 | | | 0.7711 | | | 3.3080 | | | 0.0013 |  |
| LY9 | | 1.3008 | | 0.7392 | | 3.3585 | | | 0.0011 |  | **MARCH1** | | | | 0.8535 | | | 1.6168 | | | 3.2488 | | | 0.0016 |  |
| PTCH2 | | 1.3001 | | 0.3235 | | 3.1733 | | | 0.0020 |  | **CYFIP2** | | | | 0.8497 | | | 4.6884 | | | 3.4382 | | | 0.0009 |  |
| NAPSB | | 1.2765 | | 0.9620 | | 3.5709 | | | 0.0005 |  | **CD72** | | | | 0.8481 | | | 1.3934 | | | 3.3729 | | | 0.0011 |  |
| KIAA0748 | | 1.2728 | | 0.1106 | | 3.7102 | | | 0.0003 |  | **ATP8B4** | | | | 0.8353 | | | 1.2476 | | | 3.3643 | | | 0.0011 |  |
| TLR7 | | 1.2537 | | 1.5492 | | 3.7457 | | | 0.0003 |  | **RASSF2** | | | | 0.8343 | | | 4.4597 | | | 3.2687 | | | 0.0015 |  |
| PTGDS | | 1.2351 | | 3.9529 | | 3.1019 | | | 0.0025 |  | **PLCG2** | | | | 0.7884 | | | 3.8592 | | | 3.4000 | | | 0.0010 |  |
| CD79B | | 1.2271 | | 0.9776 | | 3.6467 | | | 0.0004 |  | **DPEP2** | | | | 0.7787 | | | 0.6156 | | | 3.0821 | | | 0.0027 |  |
| CYSLTR1 | | 1.1184 | | 0.3967 | | 3.9843 | | | 0.0001 |  | **HVCN1** | | | | 0.7594 | | | 2.5669 | | | 3.3356 | | | 0.0012 |  |
| VCAM1 | | 1.0691 | | 4.4169 | | 4.4826 | | | 0.0000 |  | **SELPLG** | | | | 0.7584 | | | 4.4213 | | | 3.1234 | | | 0.0023 |  |
| RBP5 | | 1.0438 | | 0.3125 | | 3.6893 | | | 0.0004 |  | **PLCB2** | | | | 0.7506 | | | 3.3902 | | | 3.2969 | | | 0.0014 |  |
| DTX1 | | 1.0196 | | 2.2502 | | 3.6716 | | | 0.0004 |  | **ARHGEF6** | | | | 0.7480 | | | 3.9551 | | | 3.1448 | | | 0.0022 |  |
| GPNMB | | 1.0158 | | 6.5516 | | 3.2663 | | | 0.0015 |  | **LRRC33** | | | | 0.7070 | | | 2.3978 | | | 3.1490 | | | 0.0022 |  |
| LY86 | | 0.9982 | | 1.9075 | | 3.3616 | | | 0.0011 |  | **FAM113B** | | | | 0.6884 | | | 3.0357 | | | 3.1470 | | | 0.0022 |  |
| FCGR2B | | 0.9862 | | 1.7977 | | 3.2038 | | | 0.0018 |  | **ST8SIA4** | | | | 0.6288 | | | 3.4138 | | | 3.2263 | | | 0.0017 |  |
| CD28 | | 0.9735 | | 1.4648 | | 3.1560 | | | 0.0021 |  | **ARHGDIB** | | | | 0.6108 | | | 6.4449 | | | 3.1820 | | | 0.0019 |  |

**Supplementary table 2**. Eigengene value for three modules consisting of the differentially expressed genes for each patient.

| TCGA_Barcode | Moule A | Module B | Module C | Barcode | Gender | Stage | |  |
| --- | --- | --- | --- | --- | --- | --- | --- | --- |
| TCGA-3M-AB46 | -0.65838 | -0.5529 | -1.48356 | TCGA-3M-AB46 | MALE | | Stage I | |
| TCGA-3M-AB47 | 0.872611 | 1.288488 | 0.602268 | TCGA-3M-AB47 | MALE | | Stage III | |
| TCGA-B7-5816 | 0.437267 | 0.615832 | 0.204376 | TCGA-B7-5816 | FEMALE | | Stage II | |
| TCGA-B7-5818 | -1.52917 | -1.19476 | -0.04572 | TCGA-B7-5818 | MALE | | Stage I | |
| TCGA-B7-A5TI | -0.13319 | 0.214747 | -0.19943 | TCGA-B7-A5TI | MALE | | Stage III | |
| TCGA-B7-A5TJ | -1.10115 | -0.61409 | -1.07063 | TCGA-B7-A5TJ | MALE | | Stage II | |
| TCGA-B7-A5TK | 0.849508 | 0.79717 | 0.689548 | TCGA-B7-A5TK | MALE | | Stage III | |
| TCGA-B7-A5TN | 0.441262 | 0.498331 | -0.46497 | TCGA-B7-A5TN | MALE | | Stage II | |
| TCGA-BR-4187 | 1.656746 | 2.083492 | 1.600288 | TCGA-BR-4187 | MALE | | NA | |
| TCGA-BR-4191 | 0.550779 | 0.376753 | 1.305512 | TCGA-BR-4191 | MALE | | NA | |
| TCGA-BR-4201 | 1.387506 | 1.051714 | 0.683188 | TCGA-BR-4201 | FEMALE | | NA | |
| TCGA-BR-4253 | 0.095642 | -0.56503 | 1.300042 | TCGA-BR-4253 | FEMALE | | Stage III | |
| TCGA-BR-4255 | 1.414711 | 1.571451 | 0.86767 | TCGA-BR-4255 | FEMALE | | Stage III | |
| TCGA-BR-4256 | 1.235738 | 1.246774 | 1.101309 | TCGA-BR-4256 | MALE | | NA | |
| TCGA-BR-4257 | 0.333609 | -0.0176 | 0.277932 | TCGA-BR-4257 | FEMALE | | NA | |
| TCGA-BR-4267 | -0.50763 | -0.57174 | -0.10767 | TCGA-BR-4267 | MALE | | Stage I | |
| TCGA-BR-4279 | 2.151594 | 1.773632 | 1.035665 | TCGA-BR-4279 | MALE | | Stage II | |
| TCGA-BR-4280 | -1.53746 | -1.43832 | -0.39417 | TCGA-BR-4280 | FEMALE | | Stage III | |
| TCGA-BR-4292 | -1.23319 | -1.15171 | -0.0726 | TCGA-BR-4292 | FEMALE | | NA | |
| TCGA-BR-4294 | 0.785209 | -0.16537 | -0.94685 | TCGA-BR-4294 | MALE | | NA | |
| TCGA-BR-4357 | 0.490899 | 0.178836 | 0.905954 | TCGA-BR-4357 | MALE | | NA | |
| TCGA-BR-4361 | 0.671285 | 0.57952 | 0.470451 | TCGA-BR-4361 | FEMALE | | Stage III | |
| TCGA-BR-4362 | -0.5865 | -0.57528 | 0.334098 | TCGA-BR-4362 | FEMALE | | NA | |
| TCGA-BR-4363 | 0.980795 | 0.679038 | 0.636224 | TCGA-BR-4363 | FEMALE | | NA | |
| TCGA-BR-4366 | 0.636859 | -0.1582 | -0.20554 | TCGA-BR-4366 | MALE | | NA | |
| TCGA-BR-4367 | 0.639624 | 0.684708 | 0.602563 | TCGA-BR-4367 | MALE | | NA | |
| TCGA-BR-4368 | 1.094128 | 0.649344 | 0.692915 | TCGA-BR-4368 | FEMALE | | Stage IV | |
| TCGA-BR-4369 | 1.391953 | 0.388517 | 0.376633 | TCGA-BR-4369 | MALE | | NA | |
| TCGA-BR-4370 | 1.082819 | 1.062163 | 0.643333 | TCGA-BR-4370 | FEMALE | | NA | |
| TCGA-BR-4371 | -1.258 | -1.22057 | -0.81805 | TCGA-BR-4371 | FEMALE | | NA | |
| TCGA-BR-6452 | -0.40239 | -0.33929 | -0.00741 | TCGA-BR-6452 | FEMALE | | Stage II | |
| TCGA-BR-6453 | 0.547711 | 1.094971 | 2.422631 | TCGA-BR-6453 | MALE | | Stage II | |
| TCGA-BR-6454 | 0.505478 | 0.116202 | 0.668899 | TCGA-BR-6454 | MALE | | Stage II | |
| TCGA-BR-6455 | -0.40087 | -0.06811 | 0.450763 | TCGA-BR-6455 | MALE | | Stage II | |
| TCGA-BR-6456 | 1.144134 | 1.066967 | 0.374219 | TCGA-BR-6456 | FEMALE | | Stage II | |
| TCGA-BR-6457 | 1.185901 | 1.823442 | 1.067027 | TCGA-BR-6457 | MALE | | Stage II | |
| TCGA-BR-6458 | 0.582617 | 0.621662 | 0.720404 | TCGA-BR-6458 | FEMALE | | Stage II | |
| TCGA-BR-6563 | 1.470372 | 1.696999 | 1.769797 | TCGA-BR-6563 | MALE | | Stage II | |
| TCGA-BR-6564 | 1.108452 | 1.6967 | 0.738276 | TCGA-BR-6564 | FEMALE | | Stage III | |
| TCGA-BR-6565 | -0.16698 | 0.226159 | 0.32395 | TCGA-BR-6565 | MALE | | Stage II | |
| TCGA-BR-6566 | -0.62411 | -0.18687 | -0.05782 | TCGA-BR-6566 | FEMALE | | Stage II | |
| TCGA-BR-6705 | 1.638104 | 1.857592 | 0.807051 | TCGA-BR-6705 | FEMALE | | Stage III | |
| TCGA-BR-6706 | 0.751095 | 1.15225 | 1.101054 | TCGA-BR-6706 | MALE | | Stage III | |
| TCGA-BR-6707 | -0.55072 | -0.36346 | 0.398632 | TCGA-BR-6707 | MALE | | Stage II | |
| TCGA-BR-6709 | 1.200525 | 0.721649 | 1.324067 | TCGA-BR-6709 | FEMALE | | Stage III | |
| TCGA-BR-6801 | -0.54362 | 0.131487 | -1.05717 | TCGA-BR-6801 | MALE | | Stage II | |
| TCGA-BR-6802 | -0.50478 | -0.38972 | 0.26069 | TCGA-BR-6802 | MALE | | Stage III | |
| TCGA-BR-6803 | 1.058639 | 1.613408 | 1.071375 | TCGA-BR-6803 | FEMALE | | Stage II | |
| TCGA-BR-6852 | -0.26789 | 0.017165 | 0.76673 | TCGA-BR-6852 | FEMALE | | Stage II | |
| TCGA-BR-7196 | 0.965496 | 1.476992 | 1.011887 | TCGA-BR-7196 | MALE | | Stage IV | |
| TCGA-BR-7197 | -1.09008 | -0.24756 | -1.28093 | TCGA-BR-7197 | MALE | | Stage II | |
| TCGA-BR-7703 | -1.7125 | -1.18421 | -0.65771 | TCGA-BR-7703 | MALE | | Stage I | |
| TCGA-BR-7704 | -0.58572 | -0.42767 | 0.700003 | TCGA-BR-7704 | FEMALE | | Stage II | |
| TCGA-BR-7707 | -1.06345 | -0.90346 | -1.15102 | TCGA-BR-7707 | FEMALE | | Stage I | |
| TCGA-BR-7715 | -0.39894 | 0.369945 | -0.85755 | TCGA-BR-7715 | MALE | | Stage II | |
| TCGA-BR-7716 | 0.480652 | 0.193654 | 0.722599 | TCGA-BR-7716 | FEMALE | | Stage II | |
| TCGA-BR-7717 | 0.031451 | 0.215137 | -0.20383 | TCGA-BR-7717 | MALE | | Stage IV | |
| TCGA-BR-7722 | 0.07775 | 0.149314 | 0.08625 | TCGA-BR-7722 | MALE | | Stage II | |
| TCGA-BR-7723 | 0.407392 | 0.226649 | 0.376396 | TCGA-BR-7723 | MALE | | Stage III | |
| TCGA-BR-7851 | -0.44443 | -0.02856 | 0.101877 | TCGA-BR-7851 | MALE | | Stage II | |
| TCGA-BR-7901 | 0.932941 | 0.892437 | 0.242462 | TCGA-BR-7901 | MALE | | Stage II | |
| TCGA-BR-7957 | 1.290153 | 1.738256 | 0.275363 | TCGA-BR-7957 | FEMALE | | Stage IV | |
| TCGA-BR-7958 | -0.07271 | 0.226193 | 0.918658 | TCGA-BR-7958 | MALE | | Stage III | |
| TCGA-BR-7959 | 1.350304 | 1.289975 | 0.089832 | TCGA-BR-7959 | MALE | | Stage III | |
| TCGA-BR-8058 | 0.935462 | 0.945601 | 1.426808 | TCGA-BR-8058 | FEMALE | | Stage III | |
| TCGA-BR-8059 | 0.336002 | 0.718986 | -0.91422 | TCGA-BR-8059 | MALE | | Stage III | |
| TCGA-BR-8060 | 1.324913 | 0.972041 | 0.396647 | TCGA-BR-8060 | FEMALE | | Stage II | |
| TCGA-BR-8077 | -0.10169 | -0.1381 | 0.029339 | TCGA-BR-8077 | FEMALE | | Stage III | |
| TCGA-BR-8078 | -0.16369 | 0.009887 | 0.376612 | TCGA-BR-8078 | FEMALE | | Stage II | |
| TCGA-BR-8080 | 1.29067 | 1.474849 | 1.04129 | TCGA-BR-8080 | FEMALE | | Stage III | |
| TCGA-BR-8081 | 0.197161 | 0.5273 | 1.142155 | TCGA-BR-8081 | FEMALE | | Stage II | |
| TCGA-BR-8284 | 0.24301 | 0.617438 | 0.778955 | TCGA-BR-8284 | FEMALE | | Stage III | |
| TCGA-BR-8286 | 0.179871 | 0.016642 | -0.28691 | TCGA-BR-8286 | MALE | | Stage II | |
| TCGA-BR-8289 | 0.707338 | 0.534279 | -0.2264 | TCGA-BR-8289 | MALE | | Stage IV | |
| TCGA-BR-8291 | 1.161313 | 1.732546 | 1.145982 | TCGA-BR-8291 | MALE | | Stage II | |
| TCGA-BR-8295 | -0.16566 | -0.66304 | -1.30209 | TCGA-BR-8295 | FEMALE | | Stage II | |
| TCGA-BR-8296 | 0.1764 | 0.280679 | 1.251053 | TCGA-BR-8296 | FEMALE | | Stage III | |
| TCGA-BR-8297 | 0.738313 | 1.334403 | 0.16427 | TCGA-BR-8297 | MALE | | Stage III | |
| TCGA-BR-8361 | -0.88436 | -0.93953 | -0.43303 | TCGA-BR-8361 | FEMALE | | Stage III | |
| TCGA-BR-8362 | 0.378937 | 0.900507 | 0.561457 | TCGA-BR-8362 | MALE | | Stage III | |
| TCGA-BR-8363 | 0.087316 | 0.098971 | 0.592172 | TCGA-BR-8363 | FEMALE | | Stage I | |
| TCGA-BR-8364 | 1.691694 | 1.976883 | 1.111785 | TCGA-BR-8364 | FEMALE | | Stage III | |
| TCGA-BR-8365 | 1.517362 | 1.915256 | 0.92921 | TCGA-BR-8365 | FEMALE | | Stage II | |
| TCGA-BR-8366 | 0.904775 | 0.763348 | 1.407041 | TCGA-BR-8366 | FEMALE | | Stage II | |
| TCGA-BR-8367 | 0.752886 | 1.198484 | 0.130669 | TCGA-BR-8367 | MALE | | Stage III | |
| TCGA-BR-8368 | -1.58937 | -1.37615 | -0.97908 | TCGA-BR-8368 | FEMALE | | Stage I | |
| TCGA-BR-8369 | 0.400664 | 0.746964 | -0.24403 | TCGA-BR-8369 | FEMALE | | Stage III | |
| TCGA-BR-8371 | 1.134785 | 1.659965 | 1.123404 | TCGA-BR-8371 | MALE | | Stage III | |
| TCGA-BR-8372 | -0.70313 | -0.72387 | 0.342756 | TCGA-BR-8372 | MALE | | Stage III | |
| TCGA-BR-8373 | 0.362943 | 0.525761 | -0.34669 | TCGA-BR-8373 | FEMALE | | Stage III | |
| TCGA-BR-8380 | 0.97616 | 1.667955 | 0.219004 | TCGA-BR-8380 | MALE | | Stage III | |
| TCGA-BR-8381 | 0.34811 | 0.376046 | 1.064241 | TCGA-BR-8381 | MALE | | Stage II | |
| TCGA-BR-8382 | -0.35435 | 0.079936 | -0.3997 | TCGA-BR-8382 | FEMALE | | Stage III | |
| TCGA-BR-8384 | 1.368922 | 2.011108 | 0.857332 | TCGA-BR-8384 | MALE | | Stage III | |
| TCGA-BR-8483 | -0.83347 | -0.47702 | -1.75726 | TCGA-BR-8483 | MALE | | Stage III | |
| TCGA-BR-8484 | 0.558846 | 0.362857 | 0.96022 | TCGA-BR-8484 | MALE | | Stage III | |
| TCGA-BR-8485 | 0.698746 | 0.470647 | 0.086756 | TCGA-BR-8485 | FEMALE | | Stage III | |
| TCGA-BR-8486 | -0.24431 | 0.129609 | 0.062815 | TCGA-BR-8486 | FEMALE | | Stage I | |
| TCGA-BR-8487 | -1.52472 | -0.97297 | -0.67875 | TCGA-BR-8487 | FEMALE | | Stage II | |
| TCGA-BR-8588 | 0.475709 | 0.794311 | 0.758492 | TCGA-BR-8588 | FEMALE | | Stage II | |
| TCGA-BR-8589 | -1.25683 | -1.30015 | 0.421118 | TCGA-BR-8589 | MALE | | Stage III | |
| TCGA-BR-8590 | 1.03927 | 1.237841 | 1.047609 | TCGA-BR-8590 | MALE | | Stage III | |
| TCGA-BR-8591 | -0.56277 | -0.22786 | -0.10544 | TCGA-BR-8591 | MALE | | Stage III | |
| TCGA-BR-8592 | 1.458468 | 1.846804 | 1.058363 | TCGA-BR-8592 | FEMALE | | Stage III | |
| TCGA-BR-8676 | -1.5076 | -1.40287 | -0.34811 | TCGA-BR-8676 | MALE | | Stage III | |
| TCGA-BR-8677 | 0.849317 | 1.048247 | 0.483542 | TCGA-BR-8677 | FEMALE | | Stage III | |
| TCGA-BR-8678 | -0.93304 | -0.66591 | -1.41302 | TCGA-BR-8678 | MALE | | Stage I | |
| TCGA-BR-8679 | -0.58427 | 0.02973 | -0.89161 | TCGA-BR-8679 | FEMALE | | Stage I | |
| TCGA-BR-8680 | -0.66856 | -1.30803 | -1.71819 | TCGA-BR-8680 | MALE | | Stage IV | |
| TCGA-BR-8682 | 0.459456 | 1.228365 | 0.678551 | TCGA-BR-8682 | MALE | | Stage II | |
| TCGA-BR-8683 | 0.990045 | 0.675099 | 0.008366 | TCGA-BR-8683 | MALE | | Stage III | |
| TCGA-BR-8686 | 0.21661 | 0.848199 | 1.088744 | TCGA-BR-8686 | MALE | | Stage III | |
| TCGA-BR-8687 | 0.402328 | 0.181301 | -0.60318 | TCGA-BR-8687 | FEMALE | | Stage III | |
| TCGA-BR-8690 | 0.14367 | 0.338594 | 0.300713 | TCGA-BR-8690 | FEMALE | | Stage III | |
| TCGA-BR-A44T | 1.378416 | 1.332263 | 2.442518 | TCGA-BR-A44T | FEMALE | | Stage II | |
| TCGA-BR-A44U | -0.67845 | -0.0767 | -1.36933 | TCGA-BR-A44U | MALE | | Stage III | |
| TCGA-BR-A452 | -0.82539 | -0.80063 | -0.89381 | TCGA-BR-A452 | MALE | | Stage III | |
| TCGA-BR-A453 | 1.480189 | 2.141658 | 0.424007 | TCGA-BR-A453 | MALE | | Stage IV | |
| TCGA-BR-A4CR | 0.356171 | -0.37642 | -1.21667 | TCGA-BR-A4CR | FEMALE | | Stage III | |
| TCGA-BR-A4CS | -0.3194 | 0.019891 | -0.61978 | TCGA-BR-A4CS | MALE | | Stage III | |
| TCGA-BR-A4IU | 1.031494 | 1.670147 | 1.43321 | TCGA-BR-A4IU | FEMALE | | Stage III | |
| TCGA-BR-A4IV | 1.787617 | 1.995411 | 0.315819 | TCGA-BR-A4IV | MALE | | Stage III | |
| TCGA-BR-A4IY | 0.511698 | 0.436242 | 0.162131 | TCGA-BR-A4IY | MALE | | Stage II | |
| TCGA-BR-A4IZ | 1.930681 | 2.058069 | 1.076333 | TCGA-BR-A4IZ | FEMALE | | Stage III | |
| TCGA-BR-A4J1 | -0.35162 | -0.57073 | -1.63012 | TCGA-BR-A4J1 | MALE | | Stage III | |
| TCGA-BR-A4J2 | 1.485615 | 1.590057 | 1.540943 | TCGA-BR-A4J2 | MALE | | Stage II | |
| TCGA-BR-A4J4 | -0.50063 | -0.21995 | -0.15023 | TCGA-BR-A4J4 | MALE | | Stage III | |
| TCGA-BR-A4J5 | 1.096045 | 1.484062 | 0.449943 | TCGA-BR-A4J5 | MALE | | Stage III | |
| TCGA-BR-A4J6 | 0.515594 | 0.458379 | -0.48166 | TCGA-BR-A4J6 | FEMALE | | Stage II | |
| TCGA-BR-A4J7 | 1.054605 | 1.551413 | 0.875112 | TCGA-BR-A4J7 | MALE | | Stage II | |
| TCGA-BR-A4J8 | 0.008414 | 0.898967 | -0.3385 | TCGA-BR-A4J8 | FEMALE | | Stage III | |
| TCGA-BR-A4J9 | 1.274723 | 1.676511 | 0.65908 | TCGA-BR-A4J9 | MALE | | Stage II | |
| TCGA-BR-A4PD | -0.23161 | -0.43406 | 0.090598 | TCGA-BR-A4PD | FEMALE | | Stage II | |
| TCGA-BR-A4PE | -0.8897 | -0.66248 | -0.10102 | TCGA-BR-A4PE | FEMALE | | Stage I | |
| TCGA-BR-A4PF | -0.85112 | -0.74588 | -0.08665 | TCGA-BR-A4PF | MALE | | Stage III | |
| TCGA-BR-A4QI | -0.68379 | -0.54606 | -0.74552 | TCGA-BR-A4QI | FEMALE | | Stage II | |
| TCGA-BR-A4QL | -1.62575 | -1.36431 | -1.31032 | TCGA-BR-A4QL | FEMALE | | Stage III | |
| TCGA-BR-A4QM | -0.20433 | -0.05929 | 0.834512 | TCGA-BR-A4QM | MALE | | Stage III | |
| TCGA-CD-5798 | 0.955765 | 1.277277 | 0.426259 | TCGA-CD-5798 | MALE | | Stage II | |
| TCGA-CD-5799 | -0.99135 | -0.46452 | -1.32797 | TCGA-CD-5799 | MALE | | Stage II | |
| TCGA-CD-5800 | -0.38004 | -0.6627 | -0.52026 | TCGA-CD-5800 | FEMALE | | Stage II | |
| TCGA-CD-5801 | -1.07262 | -0.58843 | 0.469588 | TCGA-CD-5801 | MALE | | Stage III | |
| TCGA-CD-5803 | 1.193575 | 1.404589 | 1.783066 | TCGA-CD-5803 | FEMALE | | Stage II | |
| TCGA-CD-5804 | 0.192476 | 0.458633 | 0.442569 | TCGA-CD-5804 | MALE | | Stage III | |
| TCGA-CD-5813 | 1.453019 | 1.781417 | 1.826748 | TCGA-CD-5813 | MALE | | Stage II | |
| TCGA-CD-8524 | 0.899633 | 0.709473 | 0.070404 | TCGA-CD-8524 | FEMALE | | Stage II | |
| TCGA-CD-8525 | 0.577259 | 0.057882 | 0.205756 | TCGA-CD-8525 | FEMALE | | Stage III | |
| TCGA-CD-8526 | 0.910214 | 0.346864 | 0.162002 | TCGA-CD-8526 | FEMALE | | Stage III | |
| TCGA-CD-8527 | -0.21976 | -0.37368 | -0.91554 | TCGA-CD-8527 | FEMALE | | Stage II | |
| TCGA-CD-8528 | -1.11523 | -0.72979 | -0.90181 | TCGA-CD-8528 | FEMALE | | Stage III | |
| TCGA-CD-8529 | 0.980636 | 0.737279 | 1.465173 | TCGA-CD-8529 | MALE | | Stage IV | |
| TCGA-CD-8530 | 1.183876 | 1.417265 | 0.620483 | TCGA-CD-8530 | MALE | | Stage II | |
| TCGA-CD-8531 | -0.32147 | -0.14793 | 1.717983 | TCGA-CD-8531 | FEMALE | | Stage III | |
| TCGA-CD-8532 | 0.385966 | 0.312497 | 0.88286 | TCGA-CD-8532 | MALE | | Stage II | |
| TCGA-CD-8533 | 0.089735 | -0.00965 | -1.29467 | TCGA-CD-8533 | MALE | | Stage II | |
| TCGA-CD-8534 | 0.37696 | 0.376171 | 0.236996 | TCGA-CD-8534 | MALE | | Stage II | |
| TCGA-CD-8535 | -0.903 | -0.73482 | -1.51907 | TCGA-CD-8535 | MALE | | Stage III | |
| TCGA-CD-8536 | -0.48652 | -0.30293 | -0.4238 | TCGA-CD-8536 | MALE | | Stage II | |
| TCGA-CD-A486 | 0.308735 | -0.19181 | -0.47804 | TCGA-CD-A486 | MALE | | Stage II | |
| TCGA-CD-A487 | 0.115628 | 0.420589 | -1.13227 | TCGA-CD-A487 | MALE | | Stage II | |
| TCGA-CD-A489 | 1.729943 | 1.346115 | 0.562871 | TCGA-CD-A489 | MALE | | Stage II | |
| TCGA-CD-A48A | 0.018698 | -0.13494 | -0.43182 | TCGA-CD-A48A | MALE | | Stage II | |
| TCGA-CD-A48C | 0.558538 | 0.210478 | -0.3003 | TCGA-CD-A48C | FEMALE | | Stage II | |
| TCGA-CD-A4MG | -0.41118 | -0.08792 | 0.203134 | TCGA-CD-A4MG | MALE | | Stage II | |
| TCGA-CD-A4MH | -0.10539 | -0.20371 | -0.58999 | TCGA-CD-A4MH | FEMALE | | Stage II | |
| TCGA-CD-A4MI | 1.072268 | 1.263053 | 1.198859 | TCGA-CD-A4MI | MALE | | Stage III | |
| TCGA-CD-A4MJ | -1.44053 | -0.72587 | -1.35754 | TCGA-CD-A4MJ | MALE | | Stage I | |
| TCGA-CG-4301 | 1.7358 | 0.692236 | 0.681682 | TCGA-CG-4301 | FEMALE | | Stage IV | |
| TCGA-CG-4304 | 0.237885 | 0.319209 | 0.699398 | TCGA-CG-4304 | MALE | | Stage I | |
| TCGA-CG-4305 | 0.570989 | 0.560539 | 0.984388 | TCGA-CG-4305 | MALE | | Stage II | |
| TCGA-CG-4306 | 0.531889 | 0.096944 | 0.143316 | TCGA-CG-4306 | MALE | | Stage IV | |
| TCGA-CG-4436 | -1.62246 | -1.47124 | -0.76369 | TCGA-CG-4436 | MALE | | Stage I | |
| TCGA-CG-4437 | -0.45063 | -0.11267 | 0.504364 | TCGA-CG-4437 | MALE | | Stage II | |
| TCGA-CG-4438 | 0.844797 | -0.07666 | 0.521546 | TCGA-CG-4438 | MALE | | Stage IV | |
| TCGA-CG-4440 | 0.636406 | -0.49362 | -0.34441 | TCGA-CG-4440 | FEMALE | | Stage IV | |
| TCGA-CG-4441 | -0.28903 | 0.03278 | -0.33281 | TCGA-CG-4441 | MALE | | Stage III | |
| TCGA-CG-4442 | -0.10407 | -0.82063 | -1.00085 | TCGA-CG-4442 | MALE | | Stage I | |
| TCGA-CG-4443 | -0.89703 | -0.71964 | -0.89734 | TCGA-CG-4443 | MALE | | Stage I | |
| TCGA-CG-4444 | 0.608523 | 0.190447 | 0.640343 | TCGA-CG-4444 | MALE | | Stage III | |
| TCGA-CG-4449 | 1.386191 | 0.848581 | 0.49666 | TCGA-CG-4449 | MALE | | Stage II | |
| TCGA-CG-4460 | 1.146724 | 0.079978 | -0.02414 | TCGA-CG-4460 | FEMALE | | Stage IV | |
| TCGA-CG-4462 | 1.76476 | 1.832149 | 1.317167 | TCGA-CG-4462 | FEMALE | | Stage IV | |
| TCGA-CG-4465 | -0.09786 | -0.27524 | 0.367043 | TCGA-CG-4465 | FEMALE | | Stage IV | |
| TCGA-CG-4466 | -0.81171 | -1.24839 | -1.51167 | TCGA-CG-4466 | FEMALE | | Stage I | |
| TCGA-CG-4469 | -0.89431 | -1.11676 | -0.07005 | TCGA-CG-4469 | MALE | | Stage IV | |
| TCGA-CG-4472 | 1.623188 | 1.231265 | 1.361007 | TCGA-CG-4472 | MALE | | Stage IV | |
| TCGA-CG-4474 | 0.720854 | 0.571754 | 0.985564 | TCGA-CG-4474 | FEMALE | | Stage IV | |
| TCGA-CG-4475 | 1.420984 | 0.823611 | -0.14702 | TCGA-CG-4475 | MALE | | Stage II | |
| TCGA-CG-4476 | 1.345936 | 1.222936 | 1.242375 | TCGA-CG-4476 | MALE | | Stage III | |
| TCGA-CG-4477 | 0.727803 | 0.250264 | 0.8305 | TCGA-CG-4477 | FEMALE | | Stage I | |
| TCGA-CG-5716 | 0.128809 | -0.14514 | 0.920012 | TCGA-CG-5716 | MALE | | Stage IV | |
| TCGA-CG-5717 | 0.250967 | 0.299008 | 0.756907 | TCGA-CG-5717 | MALE | | Stage II | |
| TCGA-CG-5718 | -0.86851 | -0.71968 | 0.191613 | TCGA-CG-5718 | FEMALE | | Stage II | |
| TCGA-CG-5719 | 0.757235 | 0.937531 | 0.544499 | TCGA-CG-5719 | FEMALE | | Stage IV | |
| TCGA-CG-5720 | -0.66483 | 0.002933 | 0.595801 | TCGA-CG-5720 | MALE | | Stage I | |
| TCGA-CG-5721 | 0.324967 | -0.1671 | 1.113762 | TCGA-CG-5721 | MALE | | Stage IV | |
| TCGA-CG-5722 | 0.424201 | 0.222108 | 1.400062 | TCGA-CG-5722 | FEMALE | | Stage IV | |
| TCGA-CG-5723 | 1.00585 | -0.0021 | 0.152019 | TCGA-CG-5723 | MALE | | Stage II | |
| TCGA-CG-5724 | 0.196199 | -0.52394 | -0.61217 | TCGA-CG-5724 | MALE | | Stage IV | |
| TCGA-CG-5725 | -0.7098 | -0.77539 | -1.70097 | TCGA-CG-5725 | MALE | | Stage I | |
| TCGA-CG-5726 | -2.46291 | -1.85302 | -1.69622 | TCGA-CG-5726 | MALE | | Stage I | |
| TCGA-CG-5732 | -0.20699 | -0.47885 | 0.818289 | TCGA-CG-5732 | MALE | | Stage IV | |
| TCGA-CG-5734 | -0.00188 | -0.21682 | 1.050179 | TCGA-CG-5734 | MALE | | Stage III | |
| TCGA-D7-5577 | -0.73324 | -0.6094 | 0.629615 | TCGA-D7-5577 | FEMALE | | Stage III | |
| TCGA-D7-5578 | -0.19871 | 0.235348 | 0.643962 | TCGA-D7-5578 | MALE | | Stage III | |
| TCGA-D7-6518 | 1.525503 | 1.750429 | 0.807985 | TCGA-D7-6518 | MALE | | Stage III | |
| TCGA-D7-6519 | 0.72417 | -0.09093 | -0.36292 | TCGA-D7-6519 | FEMALE | | Stage III | |
| TCGA-D7-6520 | -0.12792 | 0.39583 | -0.35365 | TCGA-D7-6520 | MALE | | Stage III | |
| TCGA-D7-6521 | 0.9633 | 0.838826 | 1.269329 | TCGA-D7-6521 | MALE | | Stage III | |
| TCGA-D7-6522 | 1.250922 | 1.543073 | 2.435775 | TCGA-D7-6522 | MALE | | Stage I | |
| TCGA-D7-6524 | 0.557801 | 1.081278 | 0.44654 | TCGA-D7-6524 | MALE | | Stage II | |
| TCGA-D7-6525 | -0.23774 | -0.04529 | -0.4821 | TCGA-D7-6525 | MALE | | Stage III | |
| TCGA-D7-6526 | -0.31019 | -0.65487 | -0.67211 | TCGA-D7-6526 | FEMALE | | Stage III | |
| TCGA-D7-6527 | -0.26117 | -0.03814 | -0.23322 | TCGA-D7-6527 | MALE | | Stage II | |
| TCGA-D7-6528 | -2.4472 | -1.75937 | -2.03836 | TCGA-D7-6528 | FEMALE | | Stage I | |
| TCGA-D7-6815 | -0.13539 | -0.26414 | -0.49722 | TCGA-D7-6815 | FEMALE | | Stage II | |
| TCGA-D7-6817 | -0.05664 | -0.20257 | 0.38302 | TCGA-D7-6817 | MALE | | Stage III | |
| TCGA-D7-6818 | 1.240355 | 0.869576 | 0.822527 | TCGA-D7-6818 | MALE | | Stage III | |
| TCGA-D7-6820 | -1.39161 | -1.47182 | -0.88138 | TCGA-D7-6820 | MALE | | Stage II | |
| TCGA-D7-6822 | -0.67363 | -0.65473 | -0.83944 | TCGA-D7-6822 | MALE | | Stage I | |
| TCGA-D7-8570 | -0.02477 | 0.178312 | 1.33536 | TCGA-D7-8570 | MALE | | Stage III | |
| TCGA-D7-8572 | 0.838945 | 0.671952 | 0.238652 | TCGA-D7-8572 | MALE | | Stage II | |
| TCGA-D7-8573 | -2.00331 | -1.48201 | -1.18172 | TCGA-D7-8573 | MALE | | Stage II | |
| TCGA-D7-8574 | 1.105037 | 1.596337 | 2.290896 | TCGA-D7-8574 | MALE | | Stage III | |
| TCGA-D7-8575 | 0.679087 | -0.06751 | 0.543276 | TCGA-D7-8575 | MALE | | Stage III | |
| TCGA-D7-8576 | -0.5962 | -0.23902 | -0.26071 | TCGA-D7-8576 | FEMALE | | Stage III | |
| TCGA-D7-8578 | 1.06341 | 1.105082 | 0.09557 | TCGA-D7-8578 | MALE | | Stage I | |
| TCGA-D7-8579 | 0.748046 | 1.42095 | 0.735903 | TCGA-D7-8579 | FEMALE | | Stage II | |
| TCGA-D7-A4YT | -0.16359 | -0.63248 | -1.03052 | TCGA-D7-A4YT | MALE | | Stage III | |
| TCGA-D7-A4YU | 0.134337 | 0.376035 | 1.238953 | TCGA-D7-A4YU | MALE | | Stage III | |
| TCGA-D7-A4YV | -0.09355 | 0.303731 | 0.788901 | TCGA-D7-A4YV | FEMALE | | Stage II | |
| TCGA-D7-A4YX | -1.32523 | -1.1278 | -0.44342 | TCGA-D7-A4YX | MALE | | Stage II | |
| TCGA-D7-A4YY | -0.57613 | -0.26048 | -0.13529 | TCGA-D7-A4YY | MALE | | Stage III | |
| TCGA-D7-A4Z0 | 0.608575 | 0.827301 | 0.990586 | TCGA-D7-A4Z0 | FEMALE | | Stage II | |
| TCGA-D7-A6EV | -1.13972 | -1.50416 | -1.41434 | TCGA-D7-A6EV | FEMALE | | Stage II | |
| TCGA-D7-A6EX | 0.676194 | -0.03106 | -0.86229 | TCGA-D7-A6EX | FEMALE | | Stage III | |
| TCGA-D7-A6EY | 0.405997 | 0.252906 | 1.169673 | TCGA-D7-A6EY | FEMALE | | Stage III | |
| TCGA-D7-A6EZ | -1.31313 | -0.88717 | -0.19464 | TCGA-D7-A6EZ | MALE | | Stage III | |
| TCGA-D7-A6F0 | -0.13286 | -0.65022 | -0.27491 | TCGA-D7-A6F0 | FEMALE | | Stage I | |
| TCGA-D7-A6F2 | -0.40797 | -0.14981 | 0.364383 | TCGA-D7-A6F2 | MALE | | Stage I | |
| TCGA-D7-A747 | 1.087043 | 1.570781 | 1.198121 | TCGA-D7-A747 | MALE | | Stage II | |
| TCGA-D7-A748 | 1.750189 | 1.481265 | 1.566178 | TCGA-D7-A748 | FEMALE | | Stage IV | |
| TCGA-D7-A74A | -1.27197 | -1.55141 | -1.13276 | TCGA-D7-A74A | FEMALE | | Stage III | |
| TCGA-EQ-8122 | 0.154461 | 0.290303 | -0.83227 | TCGA-EQ-8122 | FEMALE | | Stage II | |
| TCGA-EQ-A4SO | -0.46299 | -1.53129 | -1.50939 | TCGA-EQ-A4SO | MALE | | Stage III | |
| TCGA-F1-6177 | -2.86369 | -2.11362 | -1.45547 | TCGA-F1-6177 | MALE | | Stage I | |
| TCGA-F1-6874 | -0.89472 | -0.44737 | -0.22959 | TCGA-F1-6874 | MALE | | Stage I | |
| TCGA-F1-6875 | -1.54298 | -1.35273 | -2.2555 | TCGA-F1-6875 | MALE | | Stage I | |
| TCGA-F1-A448 | 0.481031 | 0.371005 | 0.667092 | TCGA-F1-A448 | MALE | | Stage III | |
| TCGA-F1-A72C | 0.098935 | -0.02919 | -0.20142 | TCGA-F1-A72C | MALE | | Stage II | |
| TCGA-FP-7735 | -0.29371 | -0.17781 | 0.256351 | TCGA-FP-7735 | MALE | | Stage I | |
| TCGA-FP-7829 | -0.45289 | -0.38237 | -0.65772 | TCGA-FP-7829 | MALE | | Stage II | |
| TCGA-FP-7916 | 0.559962 | 0.764481 | 1.174865 | TCGA-FP-7916 | MALE | | Stage III | |
| TCGA-FP-7998 | 0.726836 | 0.801896 | 1.583689 | TCGA-FP-7998 | MALE | | Stage III | |
| TCGA-FP-8099 | -0.45422 | -0.27204 | -0.53391 | TCGA-FP-8099 | MALE | | Stage II | |
| TCGA-FP-8209 | 1.816155 | 2.020998 | 2.016718 | TCGA-FP-8209 | MALE | | Stage I | |
| TCGA-FP-8210 | 1.666644 | 1.863682 | 2.002611 | TCGA-FP-8210 | MALE | | Stage III | |
| TCGA-FP-8211 | 0.000702 | -0.71821 | -0.23758 | TCGA-FP-8211 | MALE | | Stage II | |
| TCGA-FP-8631 | -0.00649 | -0.06042 | -1.1205 | TCGA-FP-8631 | MALE | | Stage III | |
| TCGA-FP-A4BE | -1.39788 | -0.89127 | 0.20825 | TCGA-FP-A4BE | MALE | | Stage II | |
| TCGA-FP-A4BF | 0.848544 | 0.783721 | 0.909867 | TCGA-FP-A4BF | MALE | | Stage III | |
| TCGA-FP-A8CX | -0.56217 | -0.93189 | -0.29757 | TCGA-FP-A8CX | MALE | | Stage III | |
| TCGA-FP-A9TM | -0.17115 | -0.56806 | 1.287396 | TCGA-FP-A9TM | MALE | | Stage II | |
| TCGA-HF-7131 | -0.36044 | -0.47115 | -0.3087 | TCGA-HF-7131 | FEMALE | | Stage I | |
| TCGA-HF-7132 | 0.310053 | 0.330049 | 1.05875 | TCGA-HF-7132 | MALE | | Stage II | |
| TCGA-HF-7133 | 0.910029 | 0.073501 | 0.695041 | TCGA-HF-7133 | FEMALE | | Stage IV | |
| TCGA-HF-7134 | -1.89139 | -1.059 | -0.55095 | TCGA-HF-7134 | MALE | | Stage I | |
| TCGA-HF-7136 | 0.454836 | -0.03673 | -1.03341 | TCGA-HF-7136 | MALE | | Stage III | |
| TCGA-HF-A5NB | -1.96362 | -2.0827 | -1.35063 | TCGA-HF-A5NB | FEMALE | | Stage III | |
| TCGA-HJ-7597 | -0.74754 | -0.95379 | -0.53944 | TCGA-HJ-7597 | FEMALE | | Stage I | |
| TCGA-HU-8238 | -0.36536 | -0.37871 | 0.170887 | TCGA-HU-8238 | MALE | | Stage III | |
| TCGA-HU-8243 | -0.72922 | -0.89292 | -0.92291 | TCGA-HU-8243 | MALE | | Stage III | |
| TCGA-HU-8244 | -3.28912 | -2.70076 | -2.27449 | TCGA-HU-8244 | FEMALE | | Stage I | |
| TCGA-HU-8249 | -1.10855 | -1.03685 | -0.82208 | TCGA-HU-8249 | MALE | | Stage III | |
| TCGA-HU-8602 | -1.35304 | -1.32754 | -0.17136 | TCGA-HU-8602 | FEMALE | | Stage II | |
| TCGA-HU-8604 | -0.2766 | -0.04253 | 0.611811 | TCGA-HU-8604 | FEMALE | | Stage II | |
| TCGA-HU-8608 | -0.645 | -0.64944 | 1.106119 | TCGA-HU-8608 | MALE | | Stage III | |
| TCGA-HU-8610 | -0.87933 | -0.82054 | 0.307606 | TCGA-HU-8610 | MALE | | Stage I | |
| TCGA-HU-A4G2 | -0.96131 | -0.73977 | -0.60931 | TCGA-HU-A4G2 | MALE | | Stage II | |
| TCGA-HU-A4G3 | -0.37513 | -0.50918 | -0.61689 | TCGA-HU-A4G3 | MALE | | Stage II | |
| TCGA-HU-A4G6 | -3.44174 | -2.54903 | -1.96325 | TCGA-HU-A4G6 | MALE | | Stage I | |
| TCGA-HU-A4G8 | -0.44952 | -0.82706 | 0.133049 | TCGA-HU-A4G8 | FEMALE | | Stage II | |
| TCGA-HU-A4G9 | -3.31446 | -2.87128 | -2.55277 | TCGA-HU-A4G9 | FEMALE | | Stage I | |
| TCGA-HU-A4GC | -0.21302 | -0.17924 | -0.74467 | TCGA-HU-A4GC | MALE | | Stage III | |
| TCGA-HU-A4GD | -0.50863 | -1.09228 | -1.05205 | TCGA-HU-A4GD | MALE | | Stage II | |
| TCGA-HU-A4GF | -1.10219 | -1.17866 | -0.66437 | TCGA-HU-A4GF | MALE | | Stage II | |
| TCGA-HU-A4GH | -0.92271 | -1.66361 | -1.14595 | TCGA-HU-A4GH | MALE | | Stage I | |
| TCGA-HU-A4GJ | 1.106359 | 0.652659 | 2.704674 | TCGA-HU-A4GJ | FEMALE | | Stage III | |
| TCGA-HU-A4GN | -1.70746 | -1.44339 | -1.35819 | TCGA-HU-A4GN | MALE | | Stage II | |
| TCGA-HU-A4GP | -1.43796 | -0.68832 | -0.75111 | TCGA-HU-A4GP | FEMALE | | Stage II | |
| TCGA-HU-A4GQ | -0.55301 | 0.293968 | -1.18095 | TCGA-HU-A4GQ | MALE | | Stage III | |
| TCGA-HU-A4GT | -1.66182 | -1.4096 | -1.21742 | TCGA-HU-A4GT | FEMALE | | Stage II | |
| TCGA-HU-A4GU | -2.0193 | -1.62836 | -2.00272 | TCGA-HU-A4GU | MALE | | Stage II | |
| TCGA-HU-A4GX | -0.69841 | -0.85003 | 0.025613 | TCGA-HU-A4GX | FEMALE | | Stage III | |
| TCGA-HU-A4GY | 1.360347 | 1.481787 | 2.364313 | TCGA-HU-A4GY | FEMALE | | Stage III | |
| TCGA-HU-A4H0 | -1.69791 | -1.35104 | -0.0738 | TCGA-HU-A4H0 | MALE | | Stage III | |
| TCGA-HU-A4H2 | -0.35227 | -0.65319 | -0.29624 | TCGA-HU-A4H2 | FEMALE | | Stage III | |
| TCGA-HU-A4H3 | -1.36687 | -1.04022 | -1.01655 | TCGA-HU-A4H3 | FEMALE | | Stage III | |
| TCGA-HU-A4H4 | -0.82336 | -0.66826 | 0.271727 | TCGA-HU-A4H4 | FEMALE | | Stage II | |
| TCGA-HU-A4H5 | -1.80478 | -1.42487 | -0.99831 | TCGA-HU-A4H5 | MALE | | Stage I | |
| TCGA-HU-A4H6 | -0.06897 | -0.14214 | 0.485755 | TCGA-HU-A4H6 | FEMALE | | Stage III | |
| TCGA-HU-A4H8 | -1.92016 | -1.71797 | -1.48328 | TCGA-HU-A4H8 | MALE | | Stage I | |
| TCGA-HU-A4HB | 0.479387 | 0.075672 | 1.815559 | TCGA-HU-A4HB | MALE | | Stage II | |
| TCGA-HU-A4HD | 0.263098 | -0.06376 | -0.77371 | TCGA-HU-A4HD | MALE | | Stage III | |
| TCGA-IN-7806 | -0.91144 | -0.30182 | -0.72964 | TCGA-IN-7806 | MALE | | Stage II | |
| TCGA-IN-7808 | 0.492903 | 0.533383 | 2.351151 | TCGA-IN-7808 | MALE | | Stage III | |
| TCGA-IN-8462 | 0.700244 | 0.618708 | -0.49413 | TCGA-IN-8462 | MALE | | Stage II | |
| TCGA-IN-8663 | -0.33538 | -0.84242 | -1.73935 | TCGA-IN-8663 | MALE | | Stage II | |
| TCGA-IN-A6RI | -1.56374 | -1.91577 | -1.98797 | TCGA-IN-A6RI | MALE | | Stage I | |
| TCGA-IN-A6RJ | -1.14478 | -1.19245 | -1.39562 | TCGA-IN-A6RJ | MALE | | Stage I | |
| TCGA-IN-A6RL | 0.174201 | -0.28018 | -0.50368 | TCGA-IN-A6RL | MALE | | Stage III | |
| TCGA-IN-A6RN | 0.420166 | -0.44057 | -1.21222 | TCGA-IN-A6RN | FEMALE | | Stage III | |
| TCGA-IN-A6RO | -2.12377 | -2.17563 | -1.77732 | TCGA-IN-A6RO | MALE | | Stage I | |
| TCGA-IN-A6RR | 0.003023 | -0.75521 | -0.96757 | TCGA-IN-A6RR | MALE | | Stage III | |
| TCGA-IN-A6RS | -1.02637 | -1.47075 | -0.80238 | TCGA-IN-A6RS | MALE | | Stage I | |
| TCGA-IN-A7NR | 0.429823 | 0.160218 | 0.255485 | TCGA-IN-A7NR | FEMALE | | Stage IV | |
| TCGA-IN-A7NT | 0.028864 | -0.56481 | -1.51128 | TCGA-IN-A7NT | FEMALE | | Stage III | |
| TCGA-IN-A7NU | 0.456651 | 0.261687 | -0.04066 | TCGA-IN-A7NU | MALE | | Stage III | |
| TCGA-IN-AB1V | -0.00362 | -0.29289 | -0.71817 | TCGA-IN-AB1V | MALE | | Stage I | |
| TCGA-IN-AB1X | -0.14065 | -0.59894 | 0.095145 | TCGA-IN-AB1X | FEMALE | | Stage II | |
| TCGA-IP-7968 | 0.36945 | 0.158121 | -0.32709 | TCGA-IP-7968 | MALE | | Stage III | |
| TCGA-KB-A6F7 | -0.14655 | -1.01763 | -0.07198 | TCGA-KB-A6F7 | FEMALE | | Stage I | |
| TCGA-KB-A93G | 1.342624 | 1.206452 | 0.234044 | TCGA-KB-A93G | MALE | | Stage I | |
| TCGA-KB-A93H | -1.63594 | -1.90835 | -1.83154 | TCGA-KB-A93H | FEMALE | | Stage II | |
| TCGA-KB-A93J | -0.17028 | -0.74382 | 0.21999 | TCGA-KB-A93J | MALE | | Stage II | |
| TCGA-MX-A5UG | 1.202028 | 1.700494 | 1.829653 | TCGA-MX-A5UG | MALE | | Stage III | |
| TCGA-MX-A5UJ | 0.019705 | 0.496212 | -0.04086 | TCGA-MX-A5UJ | FEMALE | | Stage III | |
| TCGA-MX-A663 | 1.131325 | 1.367832 | 0.153828 | TCGA-MX-A663 | MALE | | Stage II | |
| TCGA-MX-A666 | 0.202218 | -0.214 | 0.586855 | TCGA-MX-A666 | MALE | | Stage II | |
| TCGA-R5-A7O7 | -0.10487 | -0.32792 | -0.47153 | TCGA-R5-A7O7 | MALE | | Stage IV | |
| TCGA-R5-A7ZF | -0.56118 | -1.43672 | -2.0465 | TCGA-R5-A7ZF | FEMALE | | Stage IV | |
| TCGA-R5-A7ZI | -0.53396 | -1.05433 | 0.632956 | TCGA-R5-A7ZI | FEMALE | | Stage IV | |
| TCGA-R5-A7ZR | -0.67245 | -1.39793 | -1.09318 | TCGA-R5-A7ZR | FEMALE | | Stage III | |
| TCGA-R5-A805 | 0.945581 | 0.065278 | 0.505885 | TCGA-R5-A805 | MALE | | Stage III | |
| TCGA-RD-A7BS | 1.269212 | 0.682894 | 0.644173 | TCGA-RD-A7BS | MALE | | Stage III | |
| TCGA-RD-A7BT | -1.14942 | -1.58247 | -1.24161 | TCGA-RD-A7BT | MALE | | Stage IV | |
| TCGA-RD-A7BW | 1.910477 | 1.807825 | 1.235859 | TCGA-RD-A7BW | FEMALE | | Stage I | |
| TCGA-RD-A7C1 | -0.46817 | -0.39267 | 0.369742 | TCGA-RD-A7C1 | MALE | | Stage I | |
| TCGA-RD-A8MV | 0.033977 | -0.17052 | 1.119639 | TCGA-RD-A8MV | MALE | | Stage III | |
| TCGA-RD-A8MW | 0.672377 | 0.448717 | 0.572339 | TCGA-RD-A8MW | MALE | | Stage III | |
| TCGA-RD-A8N0 | 1.497317 | 1.267034 | 1.886056 | TCGA-RD-A8N0 | FEMALE | | Stage III | |
| TCGA-RD-A8N1 | 0.534417 | 0.613717 | 1.570867 | TCGA-RD-A8N1 | MALE | | Stage III | |
| TCGA-RD-A8N2 | 1.42457 | 1.885496 | 0.498445 | TCGA-RD-A8N2 | FEMALE | | Stage I | |
| TCGA-RD-A8N4 | 1.094295 | 1.552884 | 0.475197 | TCGA-RD-A8N4 | FEMALE | | Stage III | |
| TCGA-RD-A8N5 | 0.954875 | 0.864451 | 0.299611 | TCGA-RD-A8N5 | MALE | | Stage III | |
| TCGA-RD-A8N6 | 0.520305 | 0.602644 | -0.75096 | TCGA-RD-A8N6 | FEMALE | | Stage III | |
| TCGA-RD-A8N9 | 1.337413 | 1.427856 | 0.882655 | TCGA-RD-A8N9 | FEMALE | | Stage II | |
| TCGA-RD-A8NB | 0.21679 | 0.644219 | 0.637186 | TCGA-RD-A8NB | FEMALE | | Stage III | |
| TCGA-SW-A7EA | -1.14163 | -0.91045 | -1.49711 | TCGA-SW-A7EA | FEMALE | | Stage I | |
| TCGA-SW-A7EB | 0.251898 | -0.06416 | 0.401399 | TCGA-SW-A7EB | MALE | | Stage III | |
| TCGA-VQ-A8DT | -0.98911 | -1.24354 | -1.30783 | TCGA-VQ-A8DT | MALE | | Stage III | |
| TCGA-VQ-A8DU | 0.551586 | 0.077337 | -0.61487 | TCGA-VQ-A8DU | MALE | | Stage III | |
| TCGA-VQ-A8DV | -0.66321 | -1.13195 | -1.74246 | TCGA-VQ-A8DV | MALE | | Stage I | |
| TCGA-VQ-A8DZ | 0.111964 | -0.34137 | -0.72309 | TCGA-VQ-A8DZ | MALE | | Stage IV | |
| TCGA-VQ-A8E0 | -0.30398 | -1.07672 | -0.75683 | TCGA-VQ-A8E0 | MALE | | Stage III | |
| TCGA-VQ-A8E2 | 0.914147 | 0.486068 | -0.7383 | TCGA-VQ-A8E2 | MALE | | Stage III | |
| TCGA-VQ-A8E3 | -1.15301 | -1.05445 | -0.54914 | TCGA-VQ-A8E3 | MALE | | Stage II | |
| TCGA-VQ-A8P2 | -1.97204 | -1.887 | -2.24397 | TCGA-VQ-A8P2 | MALE | | Stage III | |
| TCGA-VQ-A8P3 | -0.05228 | 0.006541 | -1.08106 | TCGA-VQ-A8P3 | MALE | | Stage III | |
| TCGA-VQ-A8P5 | -0.17867 | -0.13951 | -0.60741 | TCGA-VQ-A8P5 | MALE | | Stage II | |
| TCGA-VQ-A8P8 | 0.569907 | 0.674188 | 0.796742 | TCGA-VQ-A8P8 | FEMALE | | Stage II | |
| TCGA-VQ-A8PB | -0.67807 | -0.72856 | -0.89588 | TCGA-VQ-A8PB | FEMALE | | Stage II | |
| TCGA-VQ-A8PC | 0.408686 | 0.363997 | 0.9166 | TCGA-VQ-A8PC | MALE | | Stage III | |
| TCGA-VQ-A8PD | 0.460087 | 0.610285 | 1.430485 | TCGA-VQ-A8PD | MALE | | Stage III | |
| TCGA-VQ-A8PE | -0.4065 | -0.58973 | 0.097266 | TCGA-VQ-A8PE | MALE | | Stage III | |
| TCGA-VQ-A8PF | 0.083881 | 0.15747 | 1.17471 | TCGA-VQ-A8PF | MALE | | Stage III | |
| TCGA-VQ-A8PH | -0.79312 | -0.97009 | -0.34025 | TCGA-VQ-A8PH | MALE | | Stage III | |
| TCGA-VQ-A8PJ | 0.080346 | -0.8814 | -1.18736 | TCGA-VQ-A8PJ | MALE | | Stage IV | |
| TCGA-VQ-A8PK | 0.196674 | -0.35304 | -1.07062 | TCGA-VQ-A8PK | MALE | | Stage III | |
| TCGA-VQ-A8PM | 0.985259 | 0.500662 | 0.957257 | TCGA-VQ-A8PM | MALE | | Stage IV | |
| TCGA-VQ-A8PO | -1.02404 | -0.61258 | 0.326816 | TCGA-VQ-A8PO | MALE | | Stage II | |
| TCGA-VQ-A8PP | 0.502364 | -0.08025 | -0.20372 | TCGA-VQ-A8PP | MALE | | Stage IV | |
| TCGA-VQ-A8PQ | 1.20996 | 1.284062 | 2.517995 | TCGA-VQ-A8PQ | FEMALE | | Stage IV | |
| TCGA-VQ-A8PU | -1.65131 | -1.56317 | -1.59123 | TCGA-VQ-A8PU | FEMALE | | Stage III | |
| TCGA-VQ-A8PX | -1.107 | -1.6228 | -0.55007 | TCGA-VQ-A8PX | MALE | | Stage I | |
| TCGA-VQ-A91A | 0.616397 | 0.560687 | -0.02768 | TCGA-VQ-A91A | MALE | | Stage III | |
| TCGA-VQ-A91D | -1.09604 | -0.8311 | -0.76919 | TCGA-VQ-A91D | MALE | | Stage III | |
| TCGA-VQ-A91E | -1.41828 | -1.35611 | -0.09115 | TCGA-VQ-A91E | FEMALE | | Stage III | |
| TCGA-VQ-A91K | -0.79553 | -0.33135 | -0.16997 | TCGA-VQ-A91K | MALE | | Stage III | |
| TCGA-VQ-A91N | -0.09305 | -0.76814 | -0.72017 | TCGA-VQ-A91N | FEMALE | | Stage IV | |
| TCGA-VQ-A91Q | 1.372565 | 0.561946 | -0.67992 | TCGA-VQ-A91Q | MALE | | Stage IV | |
| TCGA-VQ-A91S | -0.66265 | -0.86618 | -0.53443 | TCGA-VQ-A91S | MALE | | Stage III | |
| TCGA-VQ-A91U | -1.04778 | -0.83598 | -0.18106 | TCGA-VQ-A91U | MALE | | Stage III | |
| TCGA-VQ-A91V | -0.99551 | -1.35916 | -1.5324 | TCGA-VQ-A91V | MALE | | Stage III | |
| TCGA-VQ-A91W | -1.48071 | -1.21933 | -0.07059 | TCGA-VQ-A91W | MALE | | Stage III | |
| TCGA-VQ-A91X | -3.01077 | -2.64066 | -2.16784 | TCGA-VQ-A91X | MALE | | Stage III | |
| TCGA-VQ-A91Y | 1.00964 | 1.244134 | 0.788515 | TCGA-VQ-A91Y | MALE | | Stage III | |
| TCGA-VQ-A91Z | -1.58637 | -1.91314 | -2.33926 | TCGA-VQ-A91Z | FEMALE | | Stage III | |
| TCGA-VQ-A922 | 0.97762 | 0.552038 | -0.49303 | TCGA-VQ-A922 | MALE | | Stage IV | |
| TCGA-VQ-A923 | -0.08488 | 0.125669 | 1.090088 | TCGA-VQ-A923 | MALE | | Stage III | |
| TCGA-VQ-A924 | -0.99158 | -0.70085 | -0.57774 | TCGA-VQ-A924 | MALE | | Stage II | |
| TCGA-VQ-A925 | 0.020133 | -0.33347 | -0.69751 | TCGA-VQ-A925 | MALE | | Stage III | |
| TCGA-VQ-A927 | 0.062008 | 0.119705 | -0.26991 | TCGA-VQ-A927 | MALE | | Stage III | |
| TCGA-VQ-A928 | 0.838504 | 0.367059 | -0.55339 | TCGA-VQ-A928 | MALE | | Stage IV | |
| TCGA-VQ-A92D | -1.23283 | -0.83756 | -1.83612 | TCGA-VQ-A92D | MALE | | Stage I | |
| TCGA-VQ-A94O | -0.64855 | -0.79809 | -0.90247 | TCGA-VQ-A94O | MALE | | Stage III | |
| TCGA-VQ-A94P | 1.543715 | 2.077267 | 0.680874 | TCGA-VQ-A94P | MALE | | Stage IV | |
| TCGA-VQ-A94R | 0.3526 | 0.636826 | 0.143003 | TCGA-VQ-A94R | MALE | | Stage IV | |
| TCGA-VQ-A94T | -0.59648 | -0.80238 | -1.66722 | TCGA-VQ-A94T | MALE | | Stage III | |
| TCGA-VQ-A94U | 0.121258 | 0.87172 | -0.53455 | TCGA-VQ-A94U | MALE | | Stage II | |
| TCGA-VQ-AA64 | 0.618294 | 0.401843 | -0.74543 | TCGA-VQ-AA64 | MALE | | Stage III | |
| TCGA-VQ-AA68 | -0.34592 | -0.81935 | -0.18878 | TCGA-VQ-AA68 | FEMALE | | Stage III | |
| TCGA-VQ-AA69 | -2.19769 | -2.05129 | -1.10033 | TCGA-VQ-AA69 | MALE | | Stage III | |
| TCGA-VQ-AA6A | -0.33489 | -0.91899 | -1.50113 | TCGA-VQ-AA6A | MALE | | Stage III | |
| TCGA-VQ-AA6B | -1.42609 | -0.86076 | -1.68264 | TCGA-VQ-AA6B | MALE | | Stage III | |
| TCGA-VQ-AA6D | -1.04946 | -1.51704 | -1.7824 | TCGA-VQ-AA6D | FEMALE | | Stage III | |
| TCGA-VQ-AA6F | -0.13914 | -0.36968 | 0.491499 | TCGA-VQ-AA6F | MALE | | Stage II | |
| TCGA-VQ-AA6G | -0.45016 | -1.01758 | -1.2928 | TCGA-VQ-AA6G | MALE | | Stage II | |
| TCGA-VQ-AA6I | -0.38544 | -0.73323 | -0.2249 | TCGA-VQ-AA6I | MALE | | Stage III | |
| TCGA-VQ-AA6J | -0.0765 | -0.28247 | 0.926158 | TCGA-VQ-AA6J | MALE | | Stage III | |
| TCGA-VQ-AA6K | 0.178251 | 0.205955 | -0.26589 | TCGA-VQ-AA6K | MALE | | Stage III | |
| TCGA-ZA-A8F6 | 0.388976 | 1.138777 | 0.659486 | TCGA-ZA-A8F6 | MALE | | Stage I | |
| TCGA-ZQ-A9CR | 0.680212 | 0.505944 | -0.25228 | TCGA-ZQ-A9CR | FEMALE | | Stage III | |

**Supplementary table 3**. Correlation analysis of the Eigenvalue of the three modules of the cell line provided in the GDSC database and the response of 265 drugs.

| **Drugs** | **Module A** | **Module B** | **Module C** |  | **Drugs** | **Module A** | **Module B** | **Module C** |
| --- | --- | --- | --- | --- | --- | --- | --- | --- |
| Docetaxel | -0.406 | -0.018 | 0.324 |  | A-443654 | 0.024 | 0.141 | -0.068 |
| Bleomycin (50 uM) | -0.391 | -0.095 | 0.227 |  | GW-2580 | 0.024 | -0.009 | 0.001 |
| TGX221 | -0.385 | -0.176 | 0.131 |  | Shikonin | 0.025 | -0.039 | -0.158 |
| Tanespimycin | -0.328 | 0.092 | 0.355 |  | Crizotinib | 0.027 | -0.018 | -0.025 |
| Dasatinib | -0.299 | 0.039 | 0.067 |  | JQ1 | 0.032 | 0.041 | -0.130 |
| CHIR-99021 | -0.276 | -0.191 | 0.064 |  | Rapamycin | 0.034 | 0.104 | -0.138 |
| Piperlongumine | -0.267 | -0.145 | 0.065 |  | Lenalidomide | 0.035 | 0.055 | -0.078 |
| RO-3306 | -0.265 | -0.026 | 0.189 |  | XMD11-85h | 0.036 | -0.003 | -0.107 |
| Elesclomol | -0.262 | -0.121 | 0.156 |  | BI-2536 | 0.041 | 0.169 | -0.059 |
| Trametinib | -0.256 | 0.096 | 0.202 |  | Obatoclax Mesylate | 0.041 | 0.043 | -0.149 |
| XAV939 | -0.248 | 0.078 | 0.182 |  | VX-702 | 0.043 | 0.003 | -0.085 |
| FTI-277 | -0.248 | -0.017 | 0.175 |  | OSU-03012 | 0.046 | 0.108 | -0.115 |
| JNK Inhibitor VIII | -0.241 | 0.002 | 0.217 |  | Pyrimethamine | 0.046 | 0.103 | -0.044 |
| SB216763 | -0.240 | -0.082 | 0.129 |  | JNK-9L | 0.048 | 0.078 | -0.100 |
| Midostaurin | -0.237 | -0.141 | 0.061 |  | BMS-536924 | 0.050 | 0.016 | 0.037 |
| WH-4-023 | -0.232 | -0.007 | 0.050 |  | ZG-10 | 0.055 | -0.148 | -0.190 |
| Bortezomib | -0.231 | -0.040 | 0.020 |  | Linsitinib | 0.057 | -0.002 | 0.050 |
| CHIR-99021 | -0.228 | -0.174 | 0.093 |  | Vinorelbine | 0.057 | 0.080 | -0.095 |
| Dactolisib | -0.215 | -0.090 | -0.016 |  | HG-5-88-01 | 0.059 | -0.039 | -0.054 |
| Erlotinib | -0.213 | 0.162 | 0.204 |  | Etoposide | 0.060 | -0.015 | -0.126 |
| Cisplatin | -0.209 | -0.151 | 0.090 |  | EHT-1864 | 0.063 | 0.004 | 0.016 |
| Refametinib | -0.196 | 0.070 | 0.130 |  | HG-5-113-01 | 0.064 | -0.066 | -0.129 |
| (5Z)-7-Oxozeaenol | -0.194 | -0.154 | -0.004 |  | JQ12 | 0.068 | 0.009 | -0.168 |
| Rucaparib | -0.186 | -0.165 | 0.163 |  | FMK | 0.069 | 0.049 | -0.142 |
| GSK269962A | -0.186 | -0.244 | 0.014 |  | SB52334 | 0.070 | -0.145 | -0.006 |
| Lapatinib | -0.186 | 0.227 | 0.259 |  | WIKI4 | 0.070 | -0.139 | -0.229 |
| Olaparib | -0.185 | -0.132 | 0.142 |  | Lestauritinib | 0.071 | -0.144 | -0.282 |
| PD0325901 | -0.183 | 0.116 | 0.178 |  | Pictilisib | 0.074 | 0.005 | -0.194 |
| Doramapimod | -0.178 | -0.009 | 0.167 |  | QS11 | 0.078 | 0.015 | -0.082 |
| Cetuximab | -0.176 | 0.152 | 0.177 |  | Imatinib | 0.080 | 0.012 | -0.074 |
| GSK1904529A | -0.175 | 0.041 | 0.177 |  | Sorafenib | 0.087 | 0.000 | -0.139 |
| Avagacestat | -0.174 | 0.070 | 0.203 |  | Tivozanib | 0.089 | -0.076 | -0.133 |
| Serdemetan | -0.174 | -0.081 | 0.013 |  | GSK429286A | 0.095 | -0.140 | -0.203 |
| Saracatinib | -0.174 | 0.064 | 0.085 |  | Axitinib | 0.095 | -0.099 | -0.105 |
| MG-132 | -0.173 | -0.047 | 0.047 |  | NSC-207895 | 0.096 | -0.057 | -0.136 |
| Selumetinib | -0.173 | 0.084 | 0.129 |  | CP724714 | 0.102 | 0.146 | 0.036 |
| Bicalutamide | -0.170 | -0.012 | 0.148 |  | AZD7762 | 0.103 | -0.091 | -0.306 |
| WZ-1-84 | -0.169 | 0.125 | 0.079 |  | UNC0638 | 0.104 | -0.131 | -0.224 |
| 681640 | -0.169 | 0.015 | 0.090 |  | XMD15-27 | 0.104 | -0.072 | -0.199 |
| Refametinib | -0.165 | 0.110 | 0.142 |  | GW843682X | 0.105 | 0.168 | -0.109 |
| PLX-4720 | -0.163 | -0.117 | 0.090 |  | Salubrinal | 0.107 | 0.037 | -0.187 |
| Selumetinib | -0.161 | 0.059 | 0.129 |  | Cabozantinib | 0.108 | -0.052 | -0.224 |
| Pevonedistat | -0.159 | -0.017 | 0.070 |  | Alectinib | 0.109 | -0.017 | -0.122 |
| LFM-A13 | -0.155 | -0.053 | 0.089 |  | Linifanib | 0.115 | -0.034 | -0.203 |
| GSK319347A | -0.152 | -0.139 | 0.143 |  | MK-2206 | 0.121 | 0.043 | -0.176 |
| AZD6482 | -0.151 | -0.054 | -0.047 |  | VX-11e | 0.124 | 0.132 | -0.115 |
| Bryostatin 1 | -0.150 | -0.013 | 0.086 |  | Mitomycin-C | 0.128 | 0.098 | -0.120 |
| BMS-509744 | -0.148 | 0.023 | 0.124 |  | Tozasertib | 0.129 | 0.090 | -0.141 |
| A-770041 | -0.139 | 0.099 | -0.045 |  | IPA-3 | 0.131 | -0.063 | -0.317 |
| Dabrafenib | -0.139 | -0.114 | 0.009 |  | Ruxolitinib | 0.133 | 0.008 | -0.141 |
| TW 37 | -0.137 | -0.178 | 0.009 |  | KIN001-266 | 0.134 | -0.017 | -0.199 |
| Bicalutamide | -0.136 | -0.013 | 0.111 |  | Sunitinib | 0.137 | 0.074 | -0.229 |
| Pazopanib | -0.136 | -0.180 | 0.039 |  | ZM447439 | 0.137 | -0.033 | -0.189 |
| AZD6482 | -0.135 | -0.111 | -0.071 |  | FR-180204 | 0.140 | 0.068 | -0.185 |
| PHA-665752 | -0.132 | -0.055 | 0.078 |  | Tretinoin | 0.140 | -0.034 | -0.237 |
| YK-4-279 | -0.132 | -0.067 | -0.004 |  | Entinostat | 0.143 | 0.038 | -0.155 |
| CCT007093 | -0.130 | 0.051 | 0.132 |  | STF-62247 | 0.143 | -0.019 | -0.300 |
| PF-562271 | -0.128 | 0.025 | 0.071 |  | KIN001-270 | 0.146 | -0.123 | -0.270 |
| IOX2 | -0.126 | -0.023 | 0.095 |  | Ponatinib | 0.148 | -0.053 | -0.309 |
| Embelin | -0.125 | -0.017 | 0.013 |  | BAY-61-3606 | 0.150 | -0.043 | -0.291 |
| Gefitinib | -0.122 | 0.248 | 0.145 |  | S-Trityl-L-cysteine | 0.153 | 0.112 | -0.200 |
| BMS-536924 | -0.122 | 0.108 | 0.125 |  | Nilotinib | 0.154 | 0.009 | -0.184 |
| CCT-018159 | -0.119 | 0.022 | 0.038 |  | Enzastaurin | 0.165 | 0.139 | -0.218 |
| Motesanib | -0.118 | -0.108 | 0.081 |  | QL-XII-47 | 0.165 | -0.009 | -0.267 |
| UNC1215 | -0.115 | -0.029 | 0.112 |  | Quizartinib | 0.169 | -0.044 | -0.220 |
| NSC-87877 | -0.113 | 0.017 | 0.148 |  | QL-XII-61 | 0.174 | -0.025 | -0.298 |
| QL-VIII-58 | -0.112 | -0.057 | -0.036 |  | AS605240 | 0.174 | 0.083 | -0.228 |
| Epothilone B | -0.111 | 0.128 | 0.077 |  | JQ1 | 0.183 | -0.023 | -0.289 |
| CGP-60474 | -0.111 | 0.025 | -0.082 |  | T0901317 | 0.184 | -0.019 | -0.287 |
| Temsirolimus | -0.109 | -0.101 | -0.123 |  | Y-39983 | 0.185 | -0.118 | -0.369 |
| Veliparib | -0.108 | 0.028 | 0.068 |  | VNLG/124 | 0.186 | -0.018 | -0.317 |
| Talazoparib | -0.104 | -0.177 | 0.016 |  | Amuvatinib | 0.187 | -0.060 | -0.155 |
| SGC0946 | -0.099 | -0.010 | 0.112 |  | AZD8055 | 0.200 | 0.001 | -0.348 |
| Palbociclib | -0.096 | -0.079 | -0.055 |  | PAC-1 | 0.213 | 0.053 | -0.285 |
| AS601245 | -0.095 | 0.085 | 0.057 |  | Idelalisib | 0.214 | -0.014 | -0.420 |
| Cytarabine | -0.094 | -0.092 | -0.037 |  | Pelitinib | 0.231 | 0.221 | -0.239 |
| Luminespib | -0.094 | 0.028 | -0.061 |  | THZ-2-49 | 0.231 | -0.033 | -0.421 |
| HG6-64-1 | -0.091 | -0.076 | -0.126 |  | Genentech Cpd 10 | 0.238 | -0.075 | -0.335 |
| GSK269962A | -0.088 | -0.180 | -0.050 |  | Masitinib | 0.239 | -0.066 | -0.343 |
| PLX-4720 | -0.086 | -0.059 | 0.050 |  | XMD14-99 | 0.241 | -0.060 | -0.305 |
| SN-38 | -0.085 | -0.132 | -0.095 |  | OSI-930 | 0.241 | 0.008 | -0.328 |
| Pictilisib | -0.084 | 0.000 | -0.035 |  | CX-5461 | 0.242 | -0.089 | -0.324 |
| AZ628 | -0.082 | 0.019 | 0.004 |  | PI-103 | 0.251 | -0.099 | -0.423 |
| CI-1040 | -0.079 | 0.102 | 0.023 |  | BIX02189 | 0.251 | -0.044 | -0.431 |
| Bleomycin | -0.078 | 0.069 | -0.005 |  | QL-XI-92 | 0.254 | -0.096 | -0.439 |
| CGP-082996 | -0.077 | 0.142 | -0.025 |  | Daporinad | 0.256 | -0.122 | -0.332 |
| NU7441 | -0.076 | -0.040 | -0.073 |  | AICA Ribonucleotide | 0.256 | 0.133 | -0.319 |
| Afatinib | -0.076 | 0.300 | 0.156 |  | KIN001-244 | 0.259 | -0.064 | -0.424 |
| SL0101 | -0.074 | 0.061 | 0.071 |  | MPS-1-IN-1 | 0.261 | -0.015 | -0.258 |
| NVP-TAE684 | -0.073 | -0.010 | 0.092 |  | QL-X-138 | 0.262 | -0.076 | -0.385 |
| AKT inhibitor VIII | -0.071 | 0.145 | 0.138 |  | KIN001-236 | 0.264 | -0.009 | -0.410 |
| FH535 | -0.069 | 0.092 | 0.108 |  | JW-7-24-1 | 0.267 | -0.097 | -0.443 |
| Afatinib | -0.068 | 0.318 | 0.172 |  | Belinostat | 0.272 | -0.063 | -0.364 |
| Selisistat | -0.063 | -0.080 | 0.032 |  | Omipalisib | 0.272 | 0.041 | -0.370 |
| JW-7-52-1 | -0.061 | 0.094 | -0.044 |  | CAY10603 | 0.273 | -0.143 | -0.425 |
| Thapsigargin | -0.059 | 0.004 | 0.030 |  | Fedratinib | 0.274 | -0.085 | -0.436 |
| SB590885 | -0.046 | -0.023 | 0.070 |  | Ispinesib Mesylate | 0.275 | 0.008 | -0.343 |
| GW441756 | -0.044 | 0.012 | 0.019 |  | SNX-2112 | 0.277 | -0.005 | -0.416 |
| Bexarotene | -0.040 | 0.000 | -0.033 |  | TL-1-85 | 0.277 | -0.054 | -0.462 |
| Avagacestat | -0.040 | 0.007 | -0.009 |  | OSI-027 | 0.281 | -0.065 | -0.402 |
| rTRAIL | -0.038 | -0.009 | 0.033 |  | Dacinostat | 0.281 | 0.113 | -0.290 |
| CMK | -0.037 | -0.031 | -0.104 |  | UNC0638 | 0.283 | -0.107 | -0.396 |
| Bosutinib | -0.033 | 0.135 | -0.095 |  | YM201636 | 0.286 | -0.078 | -0.365 |
| Z-LLNle-CHO | -0.032 | 0.121 | -0.156 |  | KIN001-260 | 0.286 | -0.048 | -0.441 |
| Vinblastine | -0.030 | -0.094 | -0.174 |  | ZSTK474 | 0.293 | 0.038 | -0.443 |
| Olaparib | -0.030 | -0.130 | -0.025 |  | NVP-BHG712 | 0.293 | 0.012 | -0.453 |
| Seliciclib | -0.029 | -0.007 | -0.036 |  | CP466722 | 0.293 | -0.025 | -0.423 |
| Temozolomide | -0.028 | -0.022 | -0.077 |  | CUDC-101 | 0.293 | -0.019 | -0.403 |
| XMD8-92 | -0.028 | -0.020 | -0.133 |  | NG-25 | 0.296 | -0.031 | -0.483 |
| Zibotentan | -0.027 | -0.045 | 0.006 |  | BX-912 | 0.300 | -0.126 | -0.431 |
| Cyclopamine | -0.026 | 0.001 | -0.131 |  | XMD13-2 | 0.300 | -0.046 | -0.399 |
| Camptothecin | -0.023 | -0.166 | -0.162 |  | GSK1070916 | 0.301 | -0.055 | -0.351 |
| SB-505124 | -0.022 | -0.086 | 0.064 |  | TL-2-105 | 0.307 | -0.024 | -0.315 |
| Paclitaxel | -0.021 | 0.164 | -0.053 |  | GSK690693 | 0.315 | 0.034 | -0.374 |
| BX796 | -0.018 | -0.179 | -0.159 |  | Tubastatin A | 0.317 | -0.055 | -0.452 |
| Doxorubicin | -0.017 | 0.049 | -0.046 |  | Phenformin | 0.328 | 0.062 | -0.356 |
| DMOG | -0.016 | -0.045 | -0.219 |  | THZ-2-102-1 | 0.330 | -0.016 | -0.417 |
| PF-4708671 | -0.015 | 0.048 | 0.040 |  | AR-42 | 0.333 | -0.069 | -0.422 |
| XMD8-85 | -0.013 | -0.068 | -0.177 |  | TAK-715 | 0.340 | 0.098 | -0.398 |
| Vismodegib | -0.012 | -0.001 | -0.061 |  | TPCA-1 | 0.341 | -0.008 | -0.491 |
| Nutlin-3a (-) | -0.006 | -0.143 | -0.132 |  | 5-Fluorouracil | 0.342 | 0.053 | -0.404 |
| GNF-2 | -0.005 | 0.088 | 0.012 |  | BMS-345541 | 0.343 | 0.046 | -0.391 |
| BMS-754807 | -0.004 | 0.001 | 0.124 |  | PIK-93 | 0.343 | -0.036 | -0.533 |
| KU-55933 | -0.004 | 0.037 | -0.132 |  | Navitoclax | 0.347 | -0.065 | -0.365 |
| GSK650394 | 0.004 | 0.082 | -0.016 |  | AT-7519 | 0.347 | 0.097 | -0.449 |
| Sepantronium bromide | 0.005 | -0.008 | -0.147 |  | Methotrexate | 0.365 | 0.016 | -0.463 |
| WHI-P97 | 0.006 | 0.128 | -0.052 |  | Vorinostat | 0.382 | -0.026 | -0.428 |
| Tamoxifen | 0.009 | 0.020 | -0.063 |  | NPK76-II-72-1 | 0.384 | -0.030 | -0.463 |
| Gemcitabine | 0.014 | 0.027 | -0.117 |  | PHA-793887 | 0.386 | 0.051 | -0.499 |
| PFI-1 | 0.016 | -0.030 | -0.165 |  | WZ3105 | 0.393 | 0.055 | -0.410 |
| Tipifarnib | 0.018 | 0.085 | -0.071 |  | I-BET-762 | 0.394 | -0.045 | -0.560 |
| Parthenolide | 0.023 | -0.032 | -0.156 |  | AKT inhibitor VIII | 0.421 | 0.069 | -0.465 |
| PD173074 | 0.024 | -0.026 | -0.005 |  |  |  |  |  |

**Supplementary table 4.** Top 50 drugs highly correlated with eigengene according to modules.

| **Drugs** | **Module A** |  | **Drugs** | **Module B** |  | **Drugs** | **Module C** |
| --- | --- | --- | --- | --- | --- | --- | --- |
| Docetaxel | -0.406 |  | GSK269962A | -0.244 |  | I-BET-762 | -0.560 |
| Bleomycin (50 uM) | -0.391 |  | CHIR-99021 | -0.191 |  | PIK-93 | -0.533 |
| TGX221 | -0.385 |  | GSK269962A | -0.180 |  | PHA-793887 | -0.499 |
| Tanespimycin | -0.328 |  | Pazopanib | -0.180 |  | TPCA-1 | -0.491 |
| Dasatinib | -0.299 |  | BX796 | -0.179 |  | NG-25 | -0.483 |
| CHIR-99021 | -0.276 |  | TW 37 | -0.178 |  | AKT inhibitor VIII | -0.465 |
| Piperlongumine | -0.267 |  | Talazoparib | -0.177 |  | Methotrexate | -0.463 |
| RO-3306 | -0.265 |  | TGX221 | -0.176 |  | NPK76-II-72-1 | -0.463 |
| Elesclomol | -0.262 |  | CHIR-99021 | -0.174 |  | TL-1-85 | -0.462 |
| Trametinib | -0.256 |  | Camptothecin | -0.166 |  | NVP-BHG712 | -0.453 |
| XAV939 | -0.248 |  | Rucaparib | -0.165 |  | Tubastatin A | -0.452 |
| FTI-277 | -0.248 |  | (5Z)-7-Oxozeaenol | -0.154 |  | AT-7519 | -0.449 |
| JNK Inhibitor VIII | -0.241 |  | Cisplatin | -0.151 |  | JW-7-24-1 | -0.443 |
| SB216763 | -0.240 |  | ZG-10 | -0.148 |  | ZSTK474 | -0.443 |
| Midostaurin | -0.237 |  | SB52334 | -0.145 |  | KIN001-260 | -0.441 |
| WH-4-023 | -0.232 |  | Piperlongumine | -0.145 |  | QL-XI-92 | -0.439 |
| Bortezomib | -0.231 |  | Lestauritinib | -0.144 |  | Fedratinib | -0.436 |
| CHIR-99021 | -0.228 |  | CAY10603 | -0.143 |  | BIX02189 | -0.431 |
| Dactolisib | -0.215 |  | Nutlin-3a (-) | -0.143 |  | BX-912 | -0.431 |
| Erlotinib | -0.213 |  | Midostaurin | -0.141 |  | Vorinostat | -0.428 |
| Cisplatin | -0.209 |  | GSK429286A | -0.140 |  | CAY10603 | -0.425 |
| Refametinib | -0.196 |  | WIKI4 | -0.139 |  | KIN001-244 | -0.424 |
| (5Z)-7-Oxozeaenol | -0.194 |  | GSK319347A | -0.139 |  | PI-103 | -0.423 |
| Rucaparib | -0.186 |  | SN-38 | -0.132 |  | CP466722 | -0.423 |
| GSK269962A | -0.186 |  | Olaparib | -0.132 |  | AR-42 | -0.422 |
| Lapatinib | -0.186 |  | UNC0638 | -0.131 |  | THZ-2-49 | -0.421 |
| Olaparib | -0.185 |  | Olaparib | -0.130 |  | Idelalisib | -0.420 |
| PD0325901 | -0.183 |  | BX-912 | -0.126 |  | THZ-2-102-1 | -0.417 |
| Doramapimod | -0.178 |  | KIN001-270 | -0.123 |  | SNX-2112 | -0.416 |
| Cetuximab | -0.176 |  | Daporinad | -0.122 |  | KIN001-236 | -0.410 |
| GSK1904529A | -0.175 |  | Elesclomol | -0.121 |  | WZ3105 | -0.410 |
| Avagacestat | -0.174 |  | Y-39983 | -0.118 |  | 5-Fluorouracil | -0.404 |
| Serdemetan | -0.174 |  | PLX-4720 | -0.117 |  | CUDC-101 | -0.403 |
| Saracatinib | -0.174 |  | Dabrafenib | -0.114 |  | OSI-027 | -0.402 |
| MG-132 | -0.173 |  | AZD6482 | -0.111 |  | XMD13-2 | -0.399 |
| Selumetinib | -0.173 |  | Motesanib | -0.108 |  | TAK-715 | -0.398 |
| Bicalutamide | -0.170 |  | UNC0638 | -0.107 |  | UNC0638 | -0.396 |
| WZ-1-84 | -0.169 |  | Temsirolimus | -0.101 |  | BMS-345541 | -0.391 |
| 681640 | -0.169 |  | Axitinib | -0.099 |  | QL-X-138 | -0.385 |
| Refametinib | -0.165 |  | PI-103 | -0.099 |  | GSK690693 | -0.374 |
| PLX-4720 | -0.163 |  | JW-7-24-1 | -0.097 |  | Omipalisib | -0.370 |
| Selumetinib | -0.161 |  | QL-XI-92 | -0.096 |  | Y-39983 | -0.369 |
| Pevonedistat | -0.159 |  | Bleomycin (50 uM) | -0.095 |  | YM201636 | -0.365 |
| LFM-A13 | -0.155 |  | Vinblastine | -0.094 |  | Navitoclax | -0.365 |
| GSK319347A | -0.152 |  | Cytarabine | -0.092 |  | Belinostat | -0.364 |
| AZD6482 | -0.151 |  | AZD7762 | -0.091 |  | Phenformin | -0.356 |
| Bryostatin 1 | -0.150 |  | Dactolisib | -0.090 |  | GSK1070916 | -0.351 |
| BMS-509744 | -0.148 |  | CX-5461 | -0.089 |  | AZD8055 | -0.348 |
| A-770041 | -0.139 |  | SB-505124 | -0.086 |  | Ispinesib Mesylate | -0.343 |
| Dabrafenib | -0.139 |  | Fedratinib | -0.085 |  | Masitinib | -0.343 |

**Supplementary table 5.** List of genes included in Cancer gene census (CGC) among differentially expressed genes.

| Gene | Module | Tier | Hallmark | TumorType | Role |
| --- | --- | --- | --- | --- | --- |
| AKT3 | A | 2 |  | GBM | oncogene |
| CARD11 | B | 1 | Yes | DLBCL | oncogene |
| CD28 | C | 2 |  | T-cell lymphoma | oncogene |
| CD79B | C | 1 | Yes | DLBCL, WM | oncogene |
| CDH11 | A | 1 | Yes | aneurysmal bone cyst | TSG, fusion |
| CXCR4 | C | 1 | Yes | WM | oncogene |
| EBF1 | A | 1 | Yes | lipoma | TSG, fusion |
| ERG | A | 1 | Yes | Ewing sarcoma, prostate, AML | oncogene, fusion |
| FCGR2B | C | 1 | Yes | ALL | oncogene, fusion |
| GAS7 | A | 1 | Yes | AML* | fusion |
| GATA2 | B | 1 |  | AML (CML blast transformation) | oncogene |
| GLI1 | A | 2 |  | CRC, oesophagus cancer | oncogene, fusion |
| KIT | B | 1 | Yes | GIST, AML, TGCT, mastocytosis, mucosal melanoma | oncogene |
| NBEA | A | 2 |  | large intestine carcinoma, multiple myeloma |  |
| PTPN13 | B | 1 | Yes | lung, NSCLC, gastric, peritoneal carcinomatosis | TSG |
| PTPRB | B | 1 |  | angiosarcoma | TSG |
| QKI | A | 1 | yes | angiocentric glioma, colorectal cancer | oncogene, TSG |
| RET | B | 1 |  | medullary thyroid, papillary thyroid, pheochromocytoma, NSCLC, Spitzoid tumour | oncogene, fusion |
| RNF43 | B | 1 | yes | cholangiocarcinoma, ovary, pancreas | TSG |
| RSPO3 | A | 1 |  | colorectal | oncogene, fusion |
| RUNX1T1 | A | 1 | yes | AML | oncogene, TSG, fusion |
| SETBP1 | A | 1 |  | aCML, sAML, MDS/MPN-U, CMML, JMML | oncogene, fusion |
| SFRP4 | A | 1 |  | colorectal cancer, melanoma, SCC, gastric cancer, oesophageal SCC | TSG |
| TGFBR2 | A | 1 | yes | head and neck, colorectal | TSG |
| ZEB1 | A | 2 |  | CCRCC, melanoma | oncogene |
| ZNF521 | A | 1 |  | ALL | oncogene, fusion |
| ZNRF3 | B | 2 |  | colorectal cancer, adrenocortical carcinoma, gastric cancer | TSG |

**Supplementary figure 1**. Analysis of overall survival analysis by eigengene value of three modules for external dataset (GSE26253).


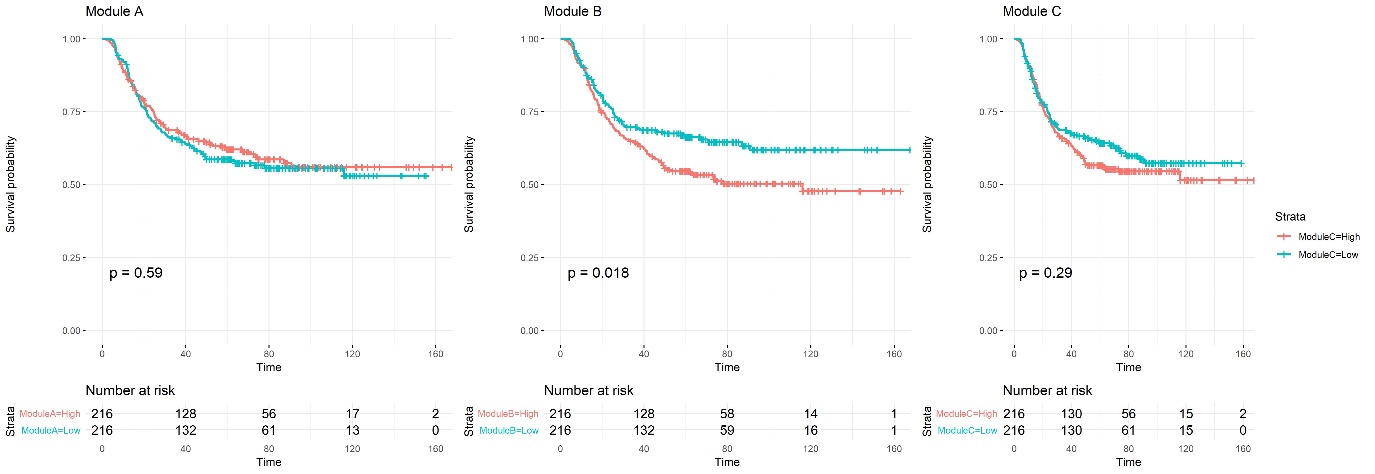

Supplement: Supplementary file 1 — Supplementary information [file 41598_2020_61016_MOESM1_ESM.docx]
